# Radio-chemotherapy and metformin selectively modulate the heterogeneous landscape of glioma with ribosome biogenesis, long non coding RNA and immune-escape markers as major player

**DOI:** 10.7150/ijbs.103194

**Published:** 2025-05-27

**Authors:** Silvia Valtorta, Silvia Granata, Stefano de Pretis, Gloria Bertoli, Serena Redaelli, Valeria Berno, Antonello E. Spinelli, Santo Diprima, Paolo Rainone, Angela Coliva, Sergio Todde, Giovanni Marfia, Stefania Navone, Manuela Caroli, Angela Bentivegna, Nadia Di Muzio, Rosa Maria Moresco

**Affiliations:** 1 Institute of Bioimaging and Complex Biological Systems (IBSBC), National Research Council (CNR), 20054 Segrate (MI), Italy;; 2 Nuclear Medicine Department, San Raffaele Scientific Institute (IRCCS), 20312 Milan, Italy;; 3 GBM-BI-TRACE (GlioBlastoMa-BIcocca-TRAnslational-CEnter), University of Milano-Bicocca, 20900 Monza, Italy;; 4 School of Medicine and Surgery, University of Milano-Bicocca, 20900 Monza, Italy;; 5 Center for Omics Sciences, San Raffaele Scientific Institute (IRCCS), 20132 Milan, Italy;; 6 NBFC, National Biodiversity Future Center, Palermo, Italy;; 7 Advanced Light and Electron Microscopy Bioimaging Center ALEMBIC, San Raffaele Scientific Institute (IRCCS), 20132 Milan, Italy;; 8 Experimental Imaging Center, San Raffaele Scientific Institute (IRCCS), 20132 Milan, Italy;; 9 Laboratory of Experimental Neurosurgery and Cell Therapy, Unit of Neurosurgery, Fondazione IRCCS Ca' Granda - Ospedale Maggiore Policlinico, 20122, Milan, Italy;; 10 Aerospace Medicine Institute “A. Mosso”, Italian Air Force, 20138, Milan, Italy;; 11 Department of Radiation Oncology, IRCCS San Raffaele Scientific Institute, 20132 Milan, Italy;; 12 Vita-Salute San Raffaele University, Milan, Italy;; 13 Fondazione IRCCS San Gerardo dei Tintori, 20900 Monza, Italy.

**Keywords:** Glioblastoma, PET imaging, single-cell RNA sequencing, LAG3, radio-chemotherapy, metformin, long-non coding RNA

## Abstract

Glioblastoma multiforme (GBM) is the most common primary brain tumor in adults with a short survival time after standard therapy administration including radiotherapy (RT) associated with temozolomide (TMZ). Here, we investigated the effects of radiochemotherapy in association with metformin (MET), a drug targeting cell metabolism on a syngeneic GBM mouse model using Positron Emission Tomography imaging with [^18^F]FLT and [^18^F]VC701 and single-cell RNA-sequencing analysis.

The addition of drugs to RT significantly increased survival and [^18^F]FLT showed an early predictive response of combined therapy. We identified the presence of heterogeneous tumor populations with different treatment sensitivity and a complex immune evasive microenvironment. Tumor cells surviving to treatments showed immune response, among the main differentially modulated biological functions and a potential role of long non-coding RNAs (lncRNAs) in treatment resistance. Association with TMZ or TMZ plus MET reduced the pro-tumor phenotype of immune reaction acting more on myeloid cells the first and on lymphocytes the latter.

Off note, MET add-on counteracted the immune-evasive phenotype particularly of T cells suggesting a potential role of MET also in adopted immunity.

## Introduction

The standard therapy for high grade glioma is based on tumor surgical resection, followed by postoperative radiotherapy (RT), and concomitant plus adjuvant Temozolomide (TMZ) 1. Despite an increase in overall survival, the prognosis remains unfavorable 2. Delayed diagnosis, infiltrative nature, cellular heterogeneity, favor treatment resistance and tumor relapse. For the reasons above, novel therapeutic approaches represent an undoubted medical need. The antineoplastic activity of the antidiabetic metformin (MET) initially emerged from epidemiological studies on type 2 diabetic patients, is manly associated with the block of mitochondrial complex I 3,4 and AMPK activation resulting in multiple cell effects including reduction of anabolic process, cell proliferation, induction of cycle arrest, and apoptosis 5. AMPK-dependent effects of MET involve the activation of different key regulators of cell homeostasis, including p53 and DICER activation as well as mTOR inhibition. Independently from AMPK, MET can modulate cancer growth and survival by reducing mTOR activity and influencing PI3K/AKT and MEK/ERK pathway. Furthermore, MET can also restore chemotherapy sensitivity by modulating NF-kB, ERK1/2 activation and autophagy. Indeed, preclinical studies including ours showed that the antidiabetic metformin exerts a synergic activity with TMZ 6-8. We previously observed that MET addition improved TMZ efficacy in glioblastoma multiforme (GBM) preclinical models with EGFR gene mutated or amplified, increasing survival time and reducing relapsing rate 9. Using PET imaging we showed that the proliferation marker 3′-deoxy-3′-[^18^F]fluorothymidine ([^18^F]FLT) was able to distinguish responder from non-responder to TMZ at early time from treatment beginning but not to predict the duration of the effect. On the contrary, the uptake of [^18^F]VC701, a radiopharmaceutical used to image neuroinflammation, thanks to its binding to the 18 kDa translocator protein (TSPO) 10 was negatively correlated with animals' survival. TMZ alone failed to reduce the inflammatory signal and, instead, increased peritumoral and infiltrating myeloid-derived suppressor cells (MDSCs), thereby promoting therapy resistance and tumor relapse 9,11. Our results indicate that both MET and TMZ reduce cell proliferation during response but also indicated an influence of MET 12 on GBM inflammatory milieu correlated with long term tumor control. Myeloid and lymphoid cells exert a central role on tumor development and escaping to treatment response 13. Recent findings on solid tumors suggest a modulatory effect of MET on these cells favoring a tumor suppressive phenotype 14. Immune system functions are clearly modulated by radiotherapy that represents a standard of care for patients affected by glioma 15. Beside the direct cytotoxic effect secondary to DNA damage, RT can influence TME. Within the tumor, RT induces a rapid increase in inflammatory response markers 16 modulating a broad spectrum of immune and stromal cells including microglia/macrophages (TAMs) and lymphocytes 17,18. Several findings in solid tumor show that cell damages, secondary to RT, induce a series of events that exert both anti- and pro-tumorigenic effects with timing and mechanisms not fully understood particularly for glioma 19,20. In addition, TMZ influences TME, not only increasing the recruitment of MDSC but also that of Regulatory T cell (Treg), thus increasing the immune suppressive effect of RT 21. Despite its relevance, data on the effect of RT given alone or in combination with chemotherapy on the GBM TME and neuro-immune response are limited. Therefore, the effect of MET add-on on GBM TME needs to be study in protocols that include RT administration. The primary aim of this study was the evaluation of MET in association with RT and TMZ on tumor growth, TME composition and immune response reprogramming. To better assess treatment effects on the GBM inflammatory milieu, the study was conducted in the GBM-immunocompetent mice model GL261 22. RT plus TMZ or plus TMZ-MET combination was first evaluated in a survival longitudinal study. Treatment effects on tumor and tumor microenvironment (TME) phenotypes were analyzed *ex vivo* in a subset of mice using fluorescence immunohistochemistry, RT-PCR, and single-cell RNA sequencing (scRNA-seq). Finally, cell proliferation and neuro-inflammation were evaluated as potential markers of early response by PET and [^18^F]FLT- or [^18^F]VC701 as radiopharmaceuticals.

## Results

### GL261 line showed genomic similarities with human brain tumor, along with the presence of distinct coexisting cell subpopulations

We first evaluated the genomic signature of GL261 to determine its similarity to human brain tumors. Copy number variations identified at mosaic levels were present only in a fraction of the sample examined, indicating a heterogeneous nature of the model consisting of different coexisting cell subpopulations. Acute treatment with TMZ or TMZ plus MET did not modify the genomic profile Figure S1. The modifications observed in GL261 were similar to syntenic chromosome regions in humans that are commonly altered in malignant glioma. A large part of chromosome 4 showed a characteristic pattern of copy-number oscillations, typically produced by chromothripsis 23, that mirrors chromosomal instability present in the syntenic human 1p region. Interestingly, chromosome 7, characterized in human glioma by copy number gain, has regions in synteny with various mouse chromosomes, all of which exhibit copy number gain. This indicates a strong genomic similarity between mouse and human lesions Table S1 for top ten regions). Specifically, we observed gains or amplifications of *Myc*, *Pdgfra*, and *Cdkn2a*, or loss of *Pten*, all of which influence cell pathways involved in cell cycle regulation, metabolic rewiring, or stress response, and are heterogeneously present in tumor cells.

### *In vitro* MET increased the effect of TMZ on proliferation and cell migration also in GL261 line

To test if GL261 cells were responsive to MET add-on, we studied *in vitro* their effect on cell cycle and survival. All treatments significantly reduced cell growth (Figure S2A) with a synergistic effect observed for MET plus TMZ (TMZ 25 µM Cell Growth Inhibition Rate: 3.6%, TMZ 25 µM+MET: 40.5%, TMZ 100 µM: 9.9%, TMZ 100 µM+MET: 41.6%) and to a minor extent for MET alone (28%) (Figure S2B). Drugs combinations exerted a higher effect on cell cycle (Figure S2C-D), as shown by the significant decrease of cyclin-dependent kinase 2 (*Cdk2*), cyclin A2 (*CycA2*) and cyclin-dependent kinase 1 (*Cdk1*) involved in G1-S and G2-M phases. All treatments reduced thymidine kinase 1 (TK1) activity, reflected by decreased [¹⁸F]FLT uptake, a marker of cell proliferation during the G1/S phase of the cell cycle (Figure S2E). The MET add-on significantly reduced pyruvate kinase 2 (PKM2) levels, while exerting either no effect or a paradoxical increase on hexokinase 2 (HK2) levels at the highest dose of TMZ (Figure S2F). Finally, MET alone or in combination inhibited cell invasion (Figure S2G-H) but not migration. Overall, these *in vitro* data confirmed the increased efficacy of the MET add-on to the TMZ treatment also on GL261 cells encouraging us to test TMZ-MET in *in vivo* experiments*.*

### In the GL261 model, radiotherapy administration increased the efficacy of TMZ with no major benefit exerted by MET adjunction during observational time

**Figure 1:**
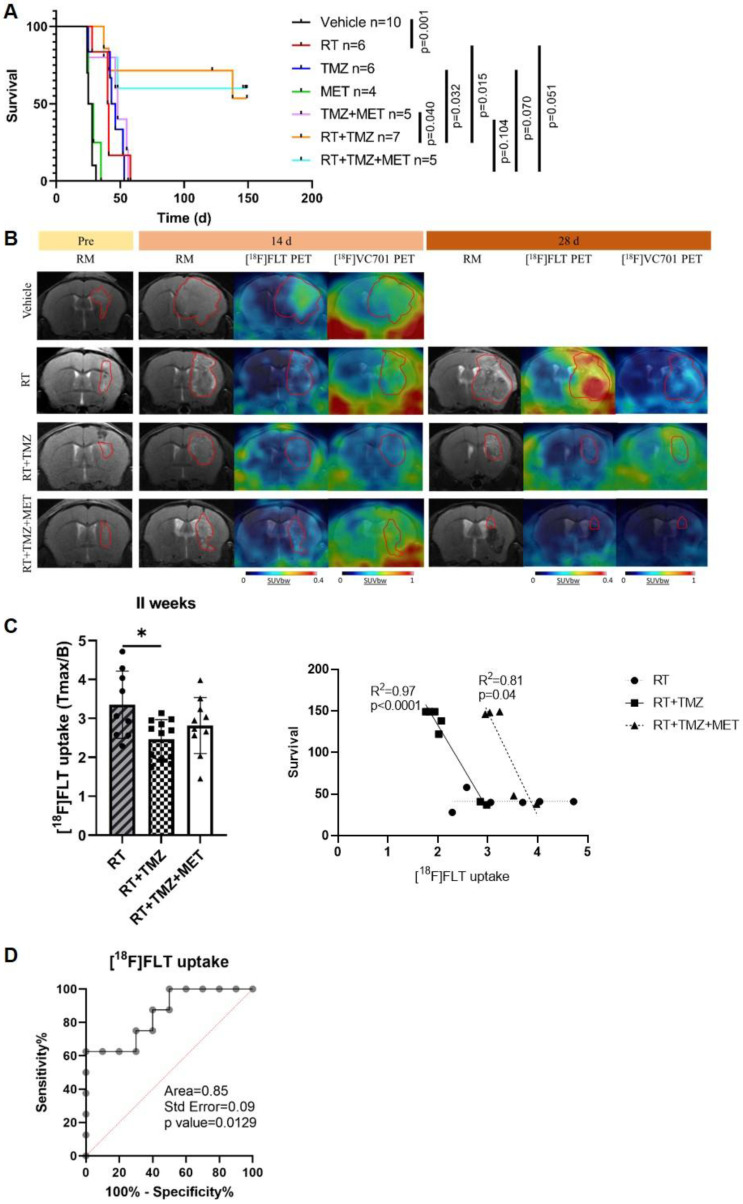
***In vivo* effect of radiotherapy treatment. A** Kaplan-Meier survival curves of GL261 bearing mice treated with vehicle, radiotherapy (RT), Temozolomide (TMZ), Metformin (MET), TMZ+MET, RT+TMZ and RT+TMZ+MET. Statistical significance was determined by the log-rank test. The *p* values are indicated. n = number of mice. **B** Representative T2-weighed MRI images and their fusion with PET images for [^18^F]FLT- and [^18^F]VC701 of GL261 bearing mice treated with vehicle, RT alone, RT+TMZ and RT+TMZ+MET acquired before the beginning of treatment and after about 14 and 28 days from the beginning of the treatment. **C** At 2 weeks we detected a significant lower uptake of [^18^F]FLT in RT+TMZ treated mice. Radiotracers uptake was expressed as tumor to background ratio. * p<0.05 by ordinary one-way ANOVA analysis followed by Tukey's multiple comparison test. Each symbol represents one animal, bars and error bars indicate group mean±sd. Correlation curve indicated that only early [^18^F]FLT uptake correlated with overall survival for both RT plus TMZ (Pearson r = -0.986, R^2^ = 0.972, p<0.0001) or RT plus TMZ and MET (Pearson r = -0.903, R^2^ = 0.816, p = 0.0356). Each symbol represents one animal. **D** ROC analysis of [^18^F]FLT uptake for prediction of different response to therapy. Optimal cut-off point was defined for [^18^F]FLT as 2.965 (75.0% sensitivity; 70.0% specificity).

We evaluated the effects of treatments, in association with RT, on mouse survival and the potential of PET to predict response. As observed in our previous studies, MET alone did not increase survival compared to vehicles (26.5 and 27 days, respectively). However, differently from mice models obtained with EGFR-mutated cells (9, in GL261, MET add-on failed to increase the effect of TMZ (48 and 44.5 days for TMZ + MET and TMZ respectively) (Figure 1A). Radiotherapy significantly prolonged the median survival time to 40.5 days, compared with vehicle-treated mice (p=0.001), approaching the survival time observed with drugs alone. Both RT plus TMZ and RT plus TMZ and MET significantly increased the time to sacrifice showing a complete response rate of approximately 60% at the end of observation time. The increased effect was visible two weeks after the beginning of the treatment as shown by Magnetic Resonance Imaging (MRI) (14 days mm^3^ Vehicle: 62.29 ± 60.98; RT: 13.73 ± 5.31; RT+TMZ: 11.46 ± 4.84; RT+TMZ+MET: 9.59 ± 7.97), and was further reinforced at 4 weeks (28 days mm^3^ RT: 21.57 ± 20.64; RT+TMZ: 6.39 ± 3.79; RT+TMZ+MET: 1.22 ± 0.81). PET showed the presence of hyper proliferative [^18^F]FLT positive lesions together with a diffuse [^18^F]VC701 uptake. [^18^F]FLT signal was localized in sub regions of T2w MRI hyper intensity whereas [^18^F]VC701 was taken up in tumor and extra tumor areas (Figure 1B and Figure S3). At 28 days only a trend over a decrease in [^18^F]FLT uptake expressed as tumor to background ratio was present in the TMZ and TMZ plus MET in comparison with RT alone (Tmax/B: RT = 5.08 + 2.32; RT+TMZ = 3.54 + 3.99; RT+TMZ+MET = 3.16 + 2.10) with no differences in [^18^F]VC701 associated radioactivity concentration. Finally, differently to what previously observed 9, early [^18^F]FLT but not [^18^F]VC701 or MRI volume, correlated with survival for both RT plus TMZ or RT plus TMZ and MET (Figure 1C). Furthermore, when mice were categorized as full responders versus non-responders or relapsed, a receiver operating characteristic (ROC) curve analysis of [¹⁸F]FLT uptake in all treated mice yielded an area under the curve (AUC) of 0.85 (p = 0.0129), with a sensitivity of 75.0% and a specificity of 70.0% at a Tmax/B cutoff value of 2.965 (Figure 1C-D). To reduce sample variability, this cutoff value was used to select RT-chemotherapy-treated animals for *ex vivo* evaluation. Overall, our results showed an increased efficacy of drugs when administered in combination with RT and an early predictive response of combined therapy only for [^18^F]FLT. However, differently to what previously observed, MET add-on failed to increase GL261 mice survival almost during the observation time.

### Radiotherapy induced a transient reduction of G1/S checkpoint markers and TK1 that was maintained over-time only by TMZ or TMZ+MET co-administration

To confirm *in vitro* findings and elucidate the effects of RT alone or in combination with TMZ or TMZ plus MET, we measured cell cycle and apoptosis-related transcript levels in separate groups of rodents immediately after RT and at 4 weeks. Early measurement of the RT effect showed a dramatic reduction in thymidine kinase 1 (TK1) levels, the [¹⁸F]FLT substrate Figure S4A), suggesting an immediate effect of irradiation on cell cycle arrest.

Consistent with PET data, the early TK1 reduction did not persist at the later time point or showed only a trend with TMZ or TMZ plus MET. Regarding the other G1/S phase markers, *CycD1*, *Cdk4*, and *Cdk6* showed an early reduction, which was maintained over time, especially with TMZ plus MET, but only for *CycD1*. *Cdk1* modulation, affecting the G2/M transition, varied across all conditions without significant effects. Overall, our data suggests a transient RT-dependent G1/S cell cycle arrest, not maintained with RT alone. RT did not modify *Bcl2*, *Bax* and *Bad* apoptosis transcripts. Association with TMZ enhanced *Bad* and *Bax* with a variable trend over an increase on *Bcl2*. MET add-on increased *Bad* and *Bcl2* transcripts, but not *Bax* (Figure S4B). All together, these data suggest a drug dependent apoptotic response in residual tumor tissue at later time after the beginning of treatment involving direct or indirect apoptotic pathway (24.

### Drugs co-administration modified the effect of RT on tumor microenvironment with a distinct effect of MET and TMZ on myeloid cell recruitment and tumor vascularization

**Figure 2:**
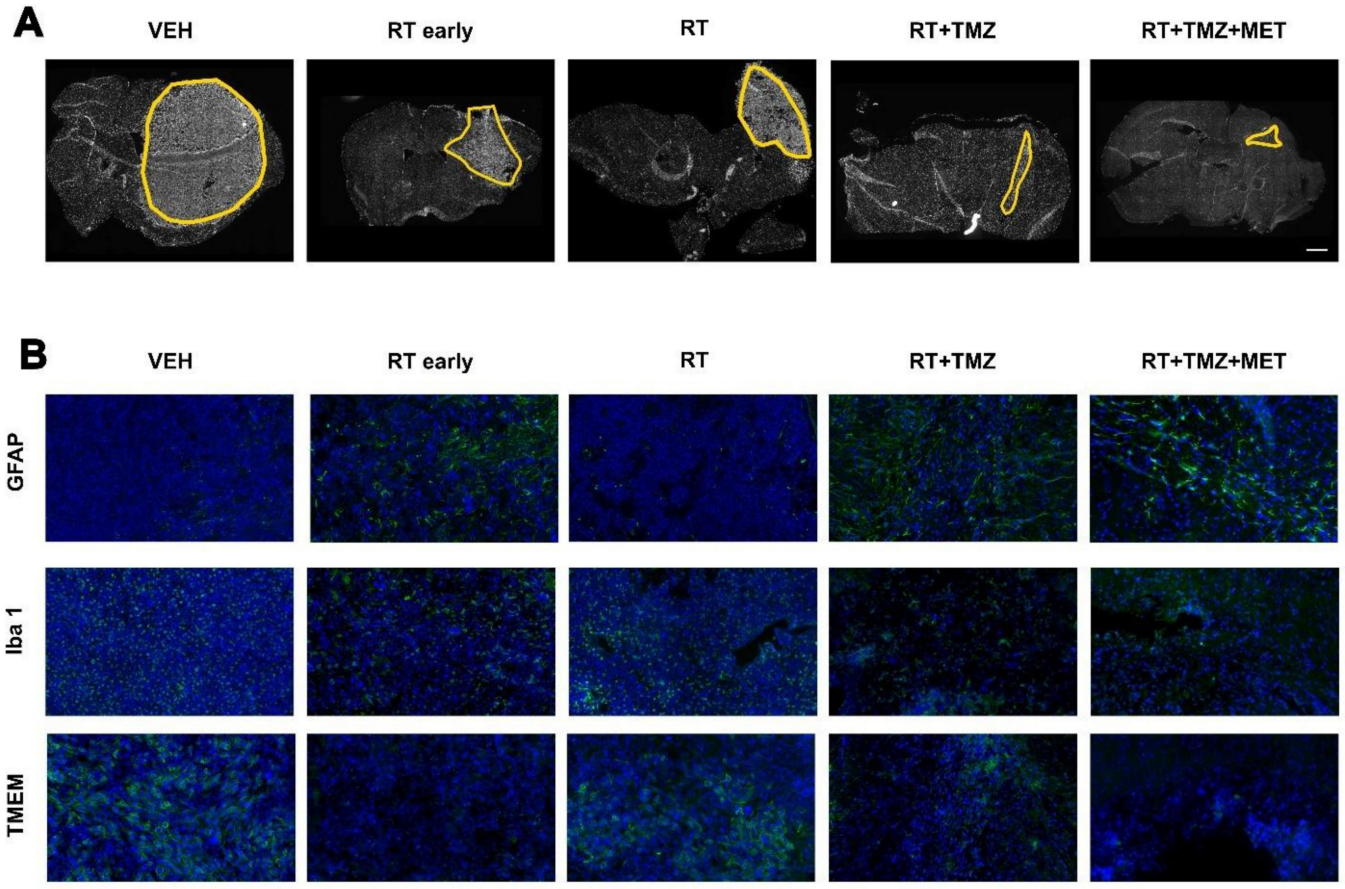
** Post treatment expression of GFAP, Iba1 and TMEM.** Immunofluorescence image of **A**) DAPI and **B**) GFAP, Iba1 and TMEM after treatment with vehicle, RT early, RT, RT+TMZ and RT+TMZ+MET. For each treatment condition, 3 samples were analyzed.

**Figure 3:**
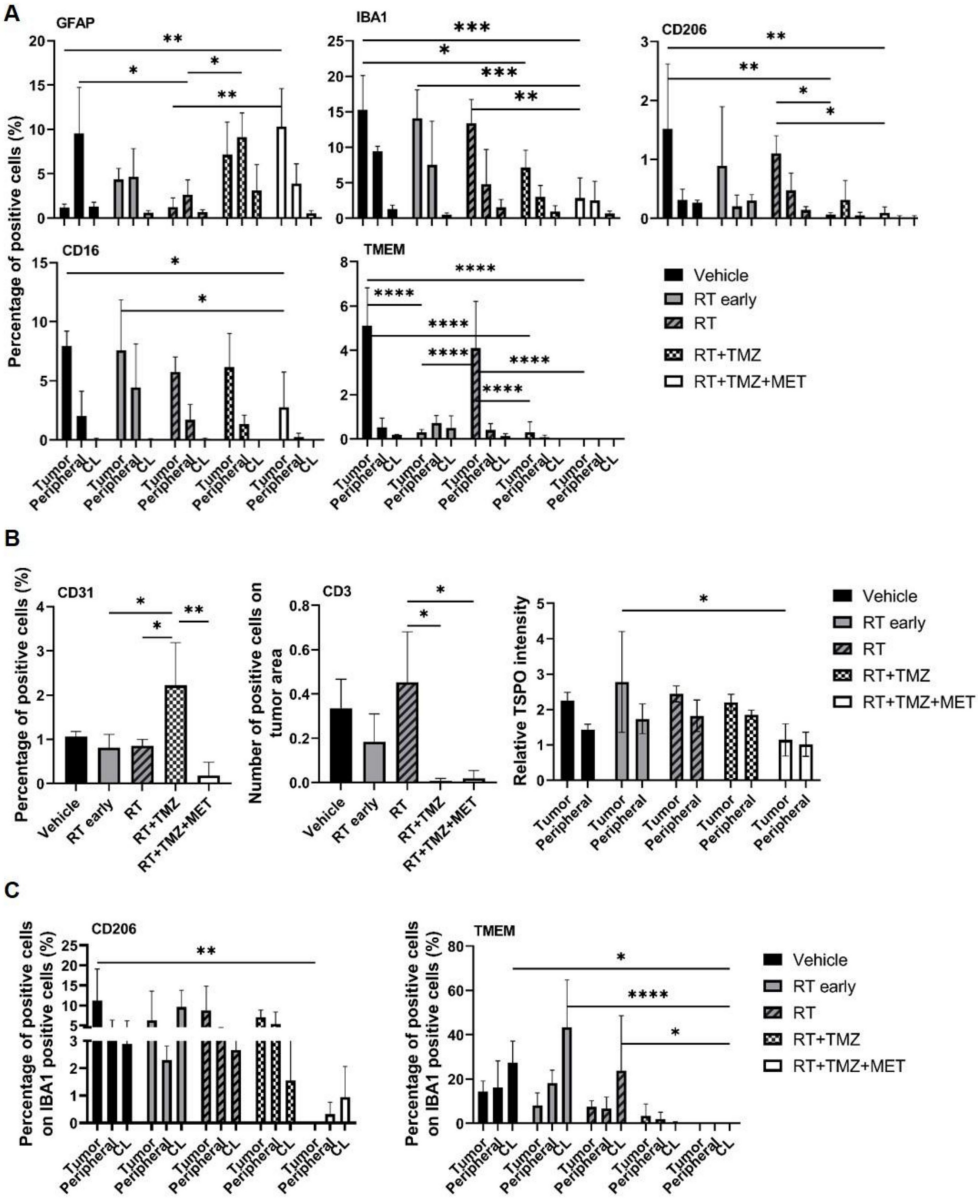
** Evaluation of RT and drugs treatment on tumor microenvironment.** Immunofluorescence image and quantification of selected markers. For each treatment condition, 3 samples were analyzed. **A**) GFAP, IBA1, CD201, CD16 and TMEM expression was quantified in the tumor (Tumor), in tumor-brain border (Peripheral) and in the brain region contralateral to the tumor (CL) and data were expressed as percentage of positive cells. **B**) CD31 expression was evaluated in all the sample and expressed as percentage of positive cells; CD3 expression was evaluated only in the tumor area and expressed as number of positive cells on tumor area and finally TSPO expression was evaluated in the tumor, in Peripheral and in CL and data expressed as ratio between the values in tumor and peripheral areas on CL. **C**) Graphs showed the expression of CD206 and TMEM on IBA1 positive cells. Data represent mean ± sd. *p<0.05, **p<0.01, ***p<0.001, ****p<0.0001 for ordinary two-way or one-way ANOVA analysis followed by Tukey's multiple comparison test.

To evaluate the treatment effect on the TME, we employed immunofluorescence to quantify the expression of general myeloid markers (TMEM119 for homeostatic microglia, IBA1 for total microglia and infiltrating peripheral myeloid cells, CD206 and CD16 for macrophages), the lymphoid marker CD3 (lymphocytes), and the astrocyte (GFAP) and vascular (CD31) markers. The effects of radiation therapy (RT) were assessed at early (5 days) and late (28 days) time points following the first administration, in three distinct regions: the tumor area, the tumor border, and the brain parenchyma contralateral to the tumor (Figure 2, 3 and figure S5). *GFAP* signal, mainly localized at the tumor border in vehicle treated animals, was reduced by RT. In the presence of drug association, an increase of intratumoral levels was observed, probably related to tissue repopulation, particularly after MET (Figure 2 and 3). In agreement with [^18^F]VC701 PET images, the neuroinflammation marker TSPO was present in both tumoral and peritumoral regions with levels minimally affected by treatment. *IBA1* immunostaining revealed predominantly amoeboid-shaped cells in lesions of vehicle- and RT-treated mice, with increased ramification observed particularly in the TMZ plus MET combination group. RT-drug combination, but not RT alone, decreased the intratumoral levels of *CD3*, *IBA1*, *CD206* and *TMEM119*, although only the last was reduced at early time. Conversely, TMEM119 co-expression on IBA1+ cells was reduced only by the drug combination, particularly MET, suggesting differential RT sensitivity of this microglial subpopulation. MET add-on selectively reduced *IBA1* cells positive for* CD206* and *CD16* populations. Finally, RT+TMZ increased tumor vascularization, an effect not observed with RT alone or in the presence of MET (Figure 2-3 and Fig. S5). Overall, immediately after the administration RT modified the intratumoral phenotype of microglial cells whereas MET add-on reduced macrophages population and counteracted the increased vascularization secondary to TMZ.

### scRNA Seq revealed a heterogeneous population of cells in GBM TME and a distinct tumor region

**Figure 4:**
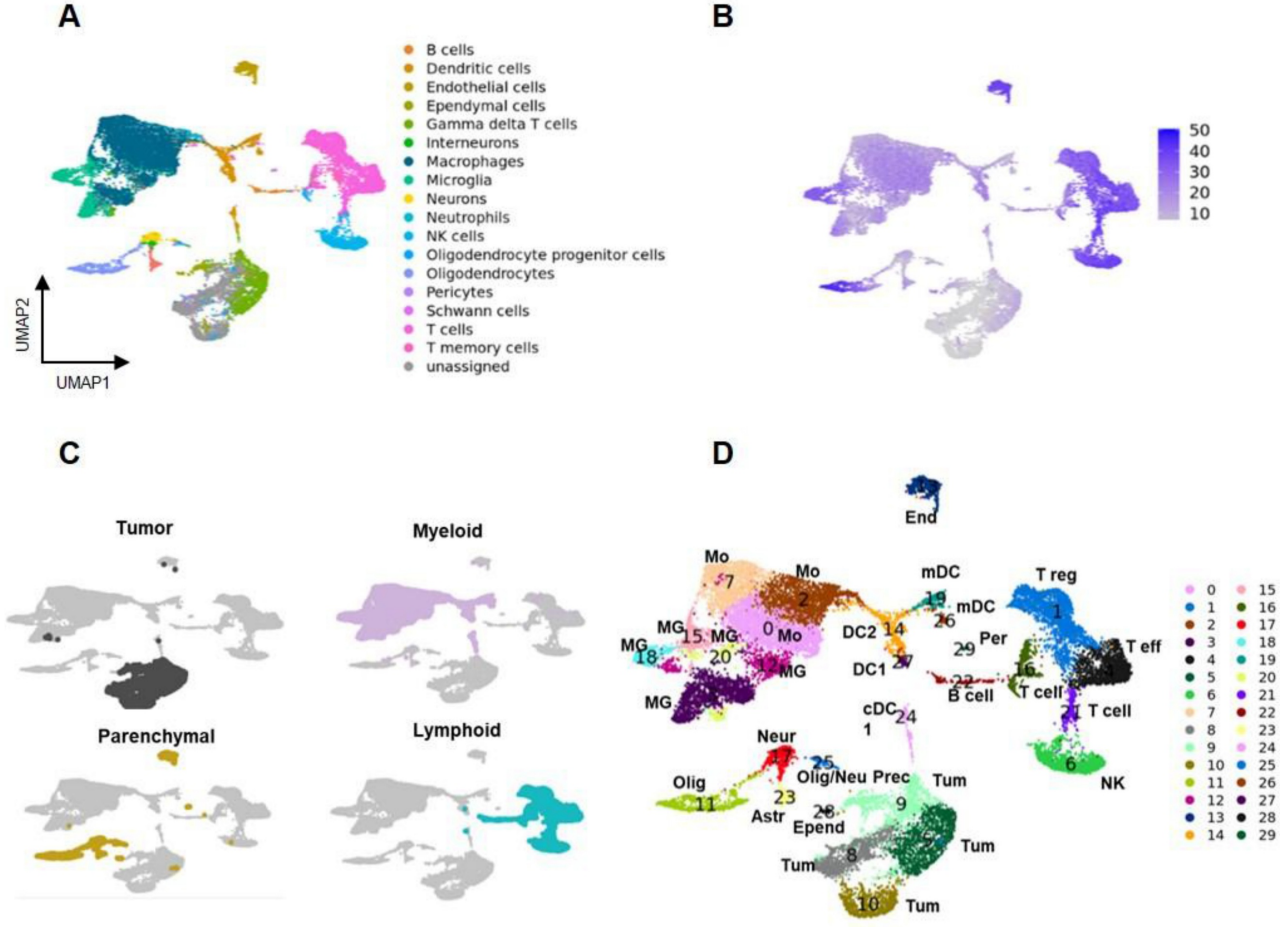
** Landscape of cells in tumor microenvironment. A** Uniform manifold approximation and projection (UMAP) plot representing cells of GBM and TME of the entire dataset obtained by merging all the samples by applying a Panglao set base panel. Each point represents one cell. **B** Genotype analysis identified a large exogenous region overlapping the unassigned area corresponding to GL261 tumor cells. **C** Tumor cells (grey), myeloid cells (pink), parenchymal cells (yellow), and lymphoid cells (light blue) were identified. **D** UMAP of the 30 clusters identified using Seurat tool. Each point represents one cells. Astr: astrocytes; Bcell: B lymphocytes; DC1: dendritic cells; DC2: dendritic cells; End: endothelial vascular cells; Epend: ependymal cells; mDC: migratory dendritic cells; MG: microglia; Mo: infiltrating monocytes; Neur: neurons; NK: natural killer cells; Olig: oligodendrocytes; Olig/neu prec: oligodendrocytes/neuron precursors; Per: pericytes; Teff: T effector lymphocytes; Treg: T regulatory lymphopcytes; Tum: tumor cells.

**Figure 5:**
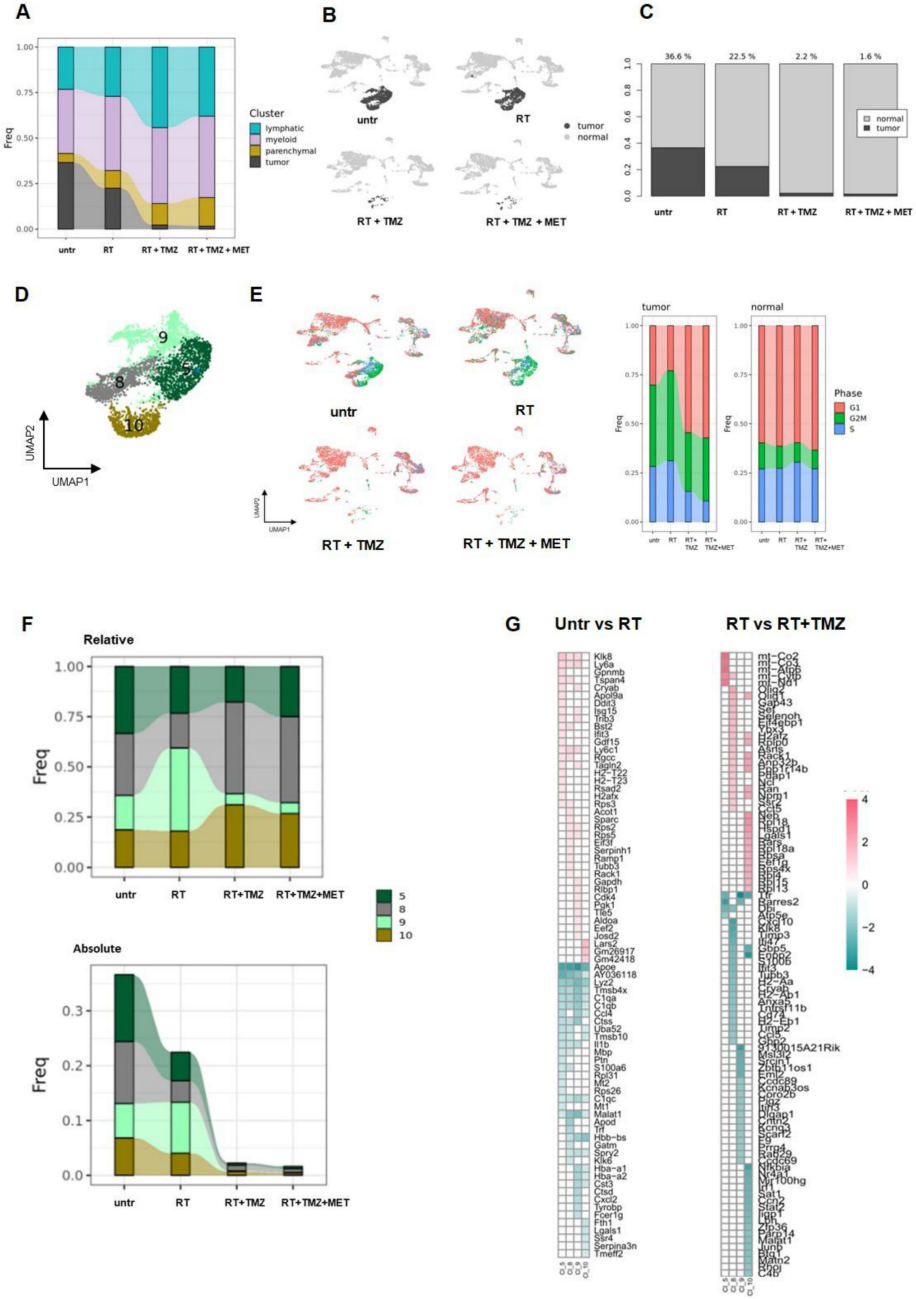
** The four tumor subclusters displayed different radio and/or chemoresistance features. A** Relative contribution of each cluster of cells in the different groups of treatment. **B** UMAP plots showing normal (light grey) and tumor (dark grey) cells. **C** Relative contribution of normal and tumor cells of UMAP plots showed in a. The numbers upon the bars indicate the percentage of tumor cells on the number of the cells of each sample. **D** UMAP plot of the tumor cluster where the 4 subclusters are showed: CL5, CL8, CL9 and CL10. **E** UMAP plots showing cells in the different cell cycle states (red: G1, green:G2M and blue: S) after treatment. The bar graphs indicated the relative contribution of tumor and normal cells in each cell cycle state. The contribution is normalized on the number of cells of each sample. **F** Relative and absolute expression of each tumor subclusters after treatment. **G** Heatmap of the common DEGs obtained from the comparison of untreated group with RT group and RT group with RT+TMZ group performed in the cluster CL5, CL8, CL9 and CL10.

To better understand the nature of GL261 lesions and the cell specific effects of treatment, a single-cell RNA-seq analysis was performed 28 days after the beginning of therapy. Cells were bi-dimensionally represented by the uniform manifold approximation and projection (UMAP) and cell-types assigned leveraging the CelliD tool coupled with the Panglao database (see methods for details). This analysis revealed heterogeneous populations of immune and parenchymal cells, along with unassigned regions (Figure 4A). Souporcell analysis identified a large region of the UMAP with an exogenous genotype that overlapped the unassigned area, corresponding to GL261 tumor cells (Figure 4B, Figure S6B-D). Unsupervised graph-based clustering using Seurat tool classified the data into 30 regions, divided into tumor, parenchymal, lymphoid, and myeloid areas (Figure 4C-D). A set of lineage associated transcript markers including *Ptprc/CD45* (myeloid and lymphoid), *Adgre1/F4-80, Itgam/CD11b* (myeloid), *Itgax/CD11c* (dendritic and myeloid) and *Cd3* (lymphoid) were used for a broad classification 25-28 Figure S6E-H, Figure S7. Clusters 3, 12, 15, 18, and 20, characterized by the expression of homeostatic microglia (MG) marker genes Tmem119, P2ry12, Sall1, and Cx3Cr1, were identified as MG. Clusters 0, 2, and 7, expressing Cxcr4, a gene associated with peripheral infiltrating monocytes (Mo)^21^, were identified as Mo. Gene transcripts for immunofluorescence (IF) markers showed the following distribution: *Fcgr3* (CD16) was present in all monocyte (Mo) clusters and microglia (MG) clusters 15, 18, and 20; *Aif1* (IBA1) was primarily detected in Mo clusters 0 and 2, and MG clusters 3 and 20; *Mrc1* (CD206) was found in Mo clusters 0 and 7. Among lymphocytes, *Cd3* expression was restricted to the T cells clusters 1, 4, 16 and 21. These cells were further subdivided into *Cd8^+^* high (cluster 4) and low (cluster 21), and *Cd4^+^* high (cluster 1) and low (cluster 16). Finally, *Gmzb* was expressed within clusters 4 and in 6. Cluster 6 also expressed *Itga2* and *CD49b* genes, confirming the CelliD classification as Natural Killer (NK) Figure S6 and Figure S7. Interestingly *Fcgr3* (CD16) was not differentially expressed by NK cells. The highest expression levels of *Tspo* were in dendritic cells (DC), followed by the MG and Mo, suggesting that PET signal was mainly associated with intra and extra tumoral inflammatory cells Figure S6H). Overall, our data showed the presence of a complex TME, representing more than 63.4% of the profiled cells. Within the TME the most enriched cells were the myeloid (35.2%) and the lymphoid (23.2%) and to a minor extent parenchymal ones (0.5%) (Figure 5A; Supplementary data to figure 5.

### Mo and MG displayed different functional phenotypes, including an immune evasive milieu

The majority of MG and Mo clusters showed high levels of M1 or M2 activation markers, often co-expressed, TAM transcripts 29-31 Figure S6F, G) and markers associated with immune evasion (32 Figure S6E and 7, Table S2. Some MG and Mo clusters resembled those described by Hochocka et al., in female tumors 33. In detail, our MG3, MG18 and MG12 exhibited partial overlaps with Hochocka's MG7, MG1 and MG2 respectively. Additionally, our Mo2 and Mo7 clusters showed similarities with monocyte/macrophage intermediate cells, while our Mo0 cluster displayed similarities with border-associated macrophages. The expression pattern suggests the presence of different cell states associated to the same phenotype Figure S8A). Both MG3 and MG15 displayed a disease associated phenotype characterized by high levels of genes involved in invasion (*Tyrobp* and *Trem2),* matrix remodeling (*Cd81*, *Spp1, Cst7, Ctsd*, *Timp2*), and complement (*C1qa, C1qb, C1qc*) mainly resembling the lipid associated and regulatory (particularly MG15) TAM according to functional classification (34. Moreover, similarly to MG18 and 20, they showed myeloid or lymphoid cell recruitment markers (see Table S3 for ligand receptor distribution). MG20 exhibited a proliferative and cytoskeleton remodeling phenotype, characterized by elevated expression of *Mki67*, *Tk1*, *Stmn1*, *H2afz*, *Pclaf,* and *Atf3*. In contrast, MG18 displayed the highest levels of homeostatic markers, resembling resident tumor-associated macrophages (TAMs) 34, while MG12 showed the lowest. Notably, MG12 expressed high levels of pro-angiogenic markers (*Cxcl2, Vegfa*), factors associated with temozolomide (TMZ) resistance (*Abc1*), and long noncoding RNAs (*Malat1*, *Xist*, and *Dleu2*) 35-38. Immune suppressive markers were particularly represented in MG18, which was the only cluster showing increased levels of the glutamine metabolism transcript *Glul*. Unlike MG, peripheral monocytes upregulated M1 and M2 markers, along with those for TAMs, pro-angiogenesis, and matrix remodeling. Immune evasion was present in all clusters, but it was particularly overexpressed in Mo7 Tables S2 and 3). Mo0 and to a lower extent Mo2 included *chemotaxis and cell migration*s and Mo7 *phagocytosis and lipid metabolism* with a profile close to lipid associated TAM (*Fabp5, ApoE and Apoc2*) (34,39,40. Mo2 displayed high levels of *Ly6c2, MHCII* and* Cxcl2, Ccl2, Cxcl9, Cxcl10* and interferon related transcripts (*Isg15*, *Ifitm3*, *Ifitm2*) with a signature similar to infiltrating myeloid derived suppressor cells (MDSCs). Within the tumor microenvironment, Mo2 may undergo transformation into either the TAM cluster Mo0 or the immune-evasive Mo7^41^ Supplementary data 2, both of which exhibit low levels of *Ly6c2*. Overall, the complexity of myeloid cells is in line with the new categorization framework, described in different solid tumors 34 but less in glioma, questioning the classical pro-anti-inflammatory dichotomy in tumor control.

### Immune escaping phenotypes involved subpopulation of dendritic cells and lymphocytes

Five different DCs clusters were identified (Figure 4D, Figure S8B, Table S2). Based on top DEG, DC24 and DC27 (*XCr1*, *Clec9a)* were classified as classical type 1 subtype 40 whereas DC14 as classical type 2 (*Ms4a4c*, *H2-aa, H2-ab1, H2-eb1* plus interferon pathway transcripts). Finally, DC19 and DC26 overexpressed *Fscn1, Ccl22* and *Ccr7*. A similar DC sub-cluster was described by Pombo Antunes et al. 40 in the same glioma model and classified as migratory or monocyte derived dendritic cells (mDC). DC19 displayed the highest levels of *Cd274/Pdl-1* transcript Supplementary data 3. DC19 and DC26, together with DC24 and DC27, showed overlapping markers Figure S8B), indicating different functional states within the same cell phenotype. DCs clusters overexpressed chemotactic markers involved in T cells recruitment (Table S2. Regarding the lymphoid region Table S4, the *Cd8*+ cluster 4 (Figure 4D, Figure S8C) showed transcripts associated with exhausted cells (*Nkg7, Cd279/Pd-1, Lag3, Ctla4, Cd27* and to a minor extent *Tox*) 42. *Cd4*+ cluster 1 was classified as Treg for the high levels of *Tnfrsf4* and *18, Icos, Ctla4, Foxp3.* Also, this cluster included *Lag3* and *Cd279/Pd-1*. Cluster 16 showed high expression of both Treg (*Foxp3*, *Tnfrsf4,18)* and T helper cell markers (*Il7r* and *Stat4)* 43, probably representing an intermediate state 44. Finally, cluster 21, displayed a naive/memory T cell signature including *Cd7*, *Vps37b, Ms4a4b, Ly6c2, Ikzf2* and *Satb1* 45 as highest expressed genes. GO analysis of top distinctive genes confirmed cluster 22 as B lymphocytes and 6 as NK Supplementary data 4. Astrocytes (cluster 23) overexpressed *Gja1*, along with transcripts involved in glycolysis (*Aldoc, Atp1a2*), neuronal remodeling (*Sparc1, Fabp7*), and a reactive phenotype (*Gfap, Aqp4*). Neurons (cluster 17) overexpressed *Snap25* and *Slc1a2*, oligodendrocytes (cluster 11) *Mog*, and oligodendrocyte precursor cells (cluster 25) *Pdgfra* and *Enpp6*. Pericytes (cluster 29) and ependymal cells (cluster 28) were characterized based on GO analysis of their top distinctive genes 46,47 Figure S8D; Supplementary data 5. Finally, a tumor endothelial signature emerged from the analysis of cluster 13 with *regulation of angiogenesis, positive regulation of cell motility, cell migration, and extracellular matrix organization* among the enriched annotations Supplementary data 5. Cluster 13 exhibited also high levels of C*xcr4, Cxcr2* and their ligand *Cxcl12,* confirming the endothelium involvement in peripheral monocyte recruitment previously described in another GBM mouse model 48.

### Tumor region was heterogeneous with highly aggressive and treatment resistant clusters

Consistent with the genomic results, the tumor region exhibited heterogeneity, dividing into four distinct clusters (Figure 5B-D). These clusters displayed varying expression levels of *Sox2*, *Sox4*, *Trp53*, and, in agreement with gene amplification analysis, *Myc* Figure S7D, Supplementary data 6. Furthermore, the clusters showed heterogeneity in cell cycle phases: CL10 was predominantly in G2/M, CL5 and CL9 in both G2/M and S, while CL8 was primarily in G1 (Figure 5E, Supplementary data 6). Notably, *Tk1*, the substrate of [^18^F]FLT, was overexpressed specifically in CL5 and CL9 Supplementary data 6, supporting the observed limitation of PET radioactivity distribution to tumor subregions. *Cell cycle* terms were enriched among the top distinctive genes and GO biological functions of all tumor clusters, except for CL10. This cluster, characterized by a high abundance of mitochondrial RNA, showed enrichment in* collagen and extracellular matrix organization* as its highest annotations. Genes associated with tumor progression, immune system regulation and apoptosis were highly expressed Figure S9A, Supplementary data 6. Specifically, *Hmgb2*, involved in DNA repair, proliferation, and apoptosis, was present in CL5 and CL9. The antiapoptotic *Birc5* was observed in CL5 and, to a lesser extent, in CL9 49,50. *Ppp1r14b* 50*,* which regulates peripheral monocyte recruitment, tumor progression, and invasion, was expressed in CL5, CL8, and CL9. Clusters 5, 8, and particularly 9, overexpressed *Lgals1* and the *Cd74* ligand *Mif,* both contributing to immune escape and TMZ resistance 51-53. Finally, the lncRNA *Malat1*, involved in tumor invasion and resistance, was overexpressed in CL10 54 , and Npm1, participating in DNA damage response, was overexpressed in CL9 55. Overall, our data showed heterogeneous populations of cycling cells with signatures favoring tumor progression and treatment resistance.

### Radio-chemotherapy reduced tumor representation showing clusters specific treatment sensitivity

**Figure 6:**
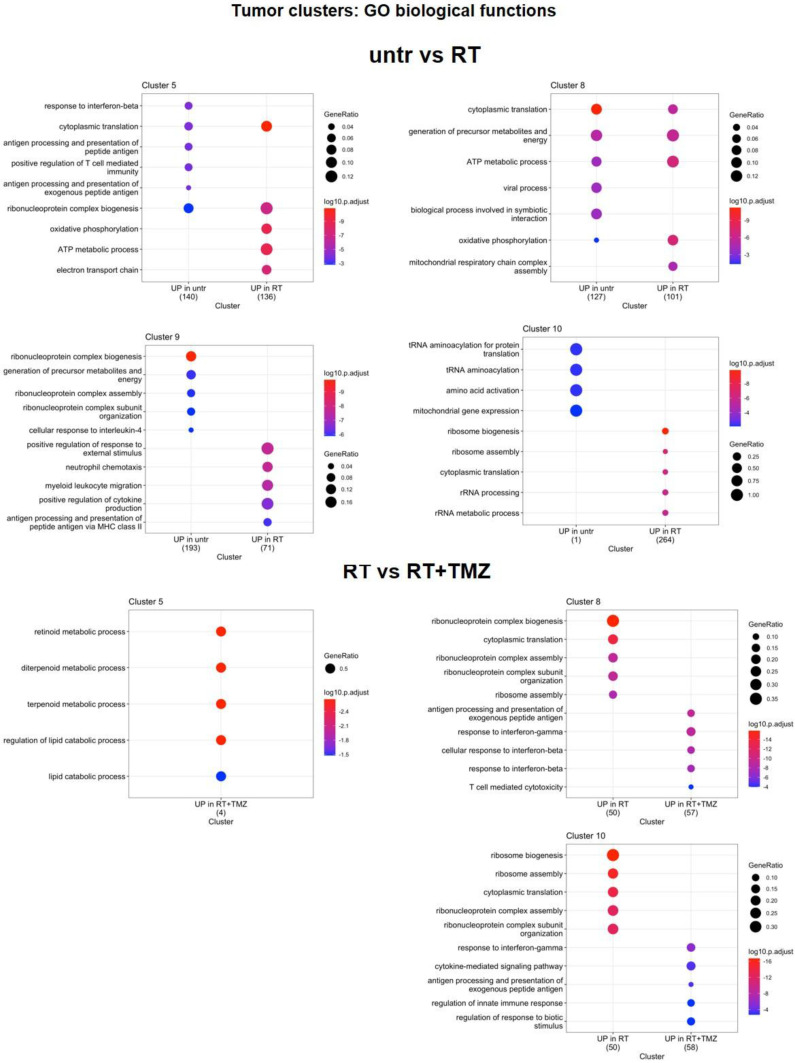
**Modification in biological functions after therapy in tumor clusters.** Top upregulated and downregulated biological functions in tumor clusters 5, 8, 9 and 10 obtained by gene ontology (GO) analysis of significant DEGs previously identified among the groups: untreated versus RT alone, RT alone versus RT plus TMZ, RT plus TMZ versus RT plus TMZ and MET.

RT partially reduced all tumor clusters except for CL9. This cluster, which showed *Mitotic G1 DNA damage checkpoint signaling* annotation and *NPM1* among its top differentially expressed transcripts Supplementary data 6, accounted for more than 40% of residual cells (Figure 5F-G). The majority of cells were in G2/M, with a lower proportion in the G1 and S phase (Figure 5E; Supplementary data 6). The add-on of TMZ alone or associated with MET improved treatment efficacy particularly for CL9. The highest fraction of residual cells was represented by CL8 (more than 40%), followed by CL10 (approximately 30%), CL5 (approximately 20%), and CL9, which accounted for less than 10% (Supplementary data to figure 5). We then applied differential gene expression (DGE) analysis to assess tumor transcriptomic changes of treatment residual cells. After RT, DGE showed a common gene signature for surviving cells with a worsening of tumor phenotype (Figure 5G, Figure S9B). All clusters upregulated *ApoE, Lyz2, C1q*, the stemness-associated *Tmsb4x* 56 and the long non-coding RNA *Malat1* 57. CL5, CL8, and CL10 upregulated the stress response transcript *Uba52* 58*,* while CL10 also upregulated the immunosuppressant *Lgals1*. The radio-resistant CL9 exhibited elevated expression of inflammatory genes (*Cxcl2*, *Il1b*, *Tyrobp*, *Ccl4*, and *Fcer1g*) and transcripts encoding complement and MHCII components. Downregulated genes were more heterogeneous, with *Klk8* and *Ly6* showing reduced expression in all clusters except CL10. At GO analysis, CL5 and CL8 upregulated annotations related to mitochondrial metabolism while reducing immune functions. Translation-related transcripts were differentially increased in CL5, CL8, and CL10, particularly those involved in translational initiation for CL5 and CL8. Conversely, the radioresistant CL9 exhibited increased chemotaxis and cytokine production, while demonstrating decreased ribonucleotide functions and cellular energy processes. Supplementary data 7, Figure 6. Overall, our results confirmed the effect of RT on ribosome assembly and activities previously described at early time on a different glioma model in all cluster except the more radioresistant one. Results indicated a persistence of the stress effect of irradiation 59, a shift in cell metabolism versus a more oxidative phenotype in CL5 and CL8 and increase in immunogenicity in the RT resistant CL9 with reduction of catabolic processes. In line with cell cycle data on the whole tissue, combined treatment with TMZ, prevented the RT dependent increase of ribosome functions and reduced the downregulation of immune response annotations (Figure 6)*.* CL8 and CL10 were the less responsive showing common overexpression of the protumor markers *Gbp5 and Enpp2* 60,61*, Cxcl10, Timp3,* and* Klk8* in CL8 and the lncRNAs *Malat 1* and* Mir100hg* in CL10 both involved in chemoresistance plus *Cd274* 62,63 Supplementary data 8. In tumor clusters no differences between TMZ or TMZ plus MET were detected.

### Radio-chemotherapy differentially modulated the immune evasive and treatment resistant milieu with the involvement of lncRNA transcripts

**Figure 7:**
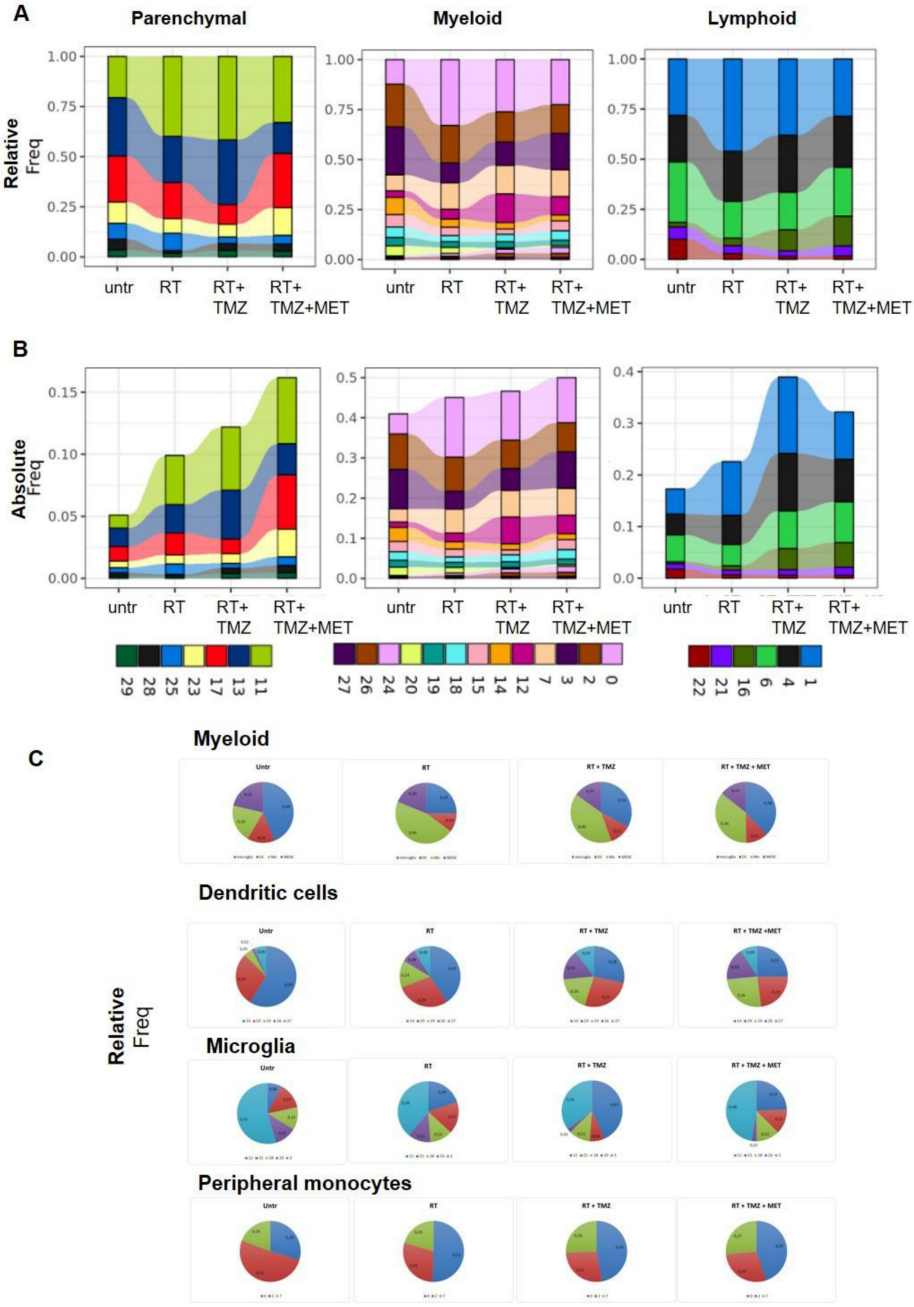
**Modulation of non-tumor cell populations after RT and chemotherapy.** The bar graphs indicate the relative **A**) and the absolute **B**) contribution of parenchymal, myeloid, and lymphoid clusters. For the relative graphs the contribution is normalized on the number of cells of each sample. Relative contribution of dendritic, microglia and peripheral monocytes in myeloid cells and in single clusters are shown in panel **C**).

The parenchymal clusters were only minimally affected by treatments. Remarkable was the decrease of the glioma associated signature of astrocytes (*Lgals3, S100 A11, Lgfbpl1* and* Nupr1*) after RT 64 and of transcripts (*Cd74, ApoE e Lyz2*) associated with neurodegeneration in oligodendrocytes 65 Supplementary data 9. All treatments modified the immune cells environment (Figure 7A, B, Tables S5, 6 and 7). RT increased peripheral monocyte representation within the myeloid cluster (Figure 5C and Figure 7A-C; Supplementary data to Figure 7), while reducing microglia, an effect that persisted after chemotherapy. MDSCs Mo2 cluster remained stable during treatment suggesting a continuous recruitment from periphery and a subsequent differentiation in TAMs. Microglia showed a relative decrease of MG3 after RT. In line with the increased vascularization marker observed at IF, the pro angiogenesis MG12 cluster was increased after TMZ. Finally, the cycling MG20 was highly reduced after drug association. Absolute levels of DCs were not modified except for cDC2 cluster 14 that was reduced by all treatments with a relative representation decreased by drugs add-on. Furthermore, radiotherapy alone or associated with TMZ increased the relative abundance of Treg, an effect not observed with MET (Figure 7A-B, Supplementary data to Figure 7). The less activated cluster 16 was more represented in the presence of chemotherapy suggesting a recent recruitment, whereas a decrement of B cells was observed (Figure 7A, B).

**Figure 8:**
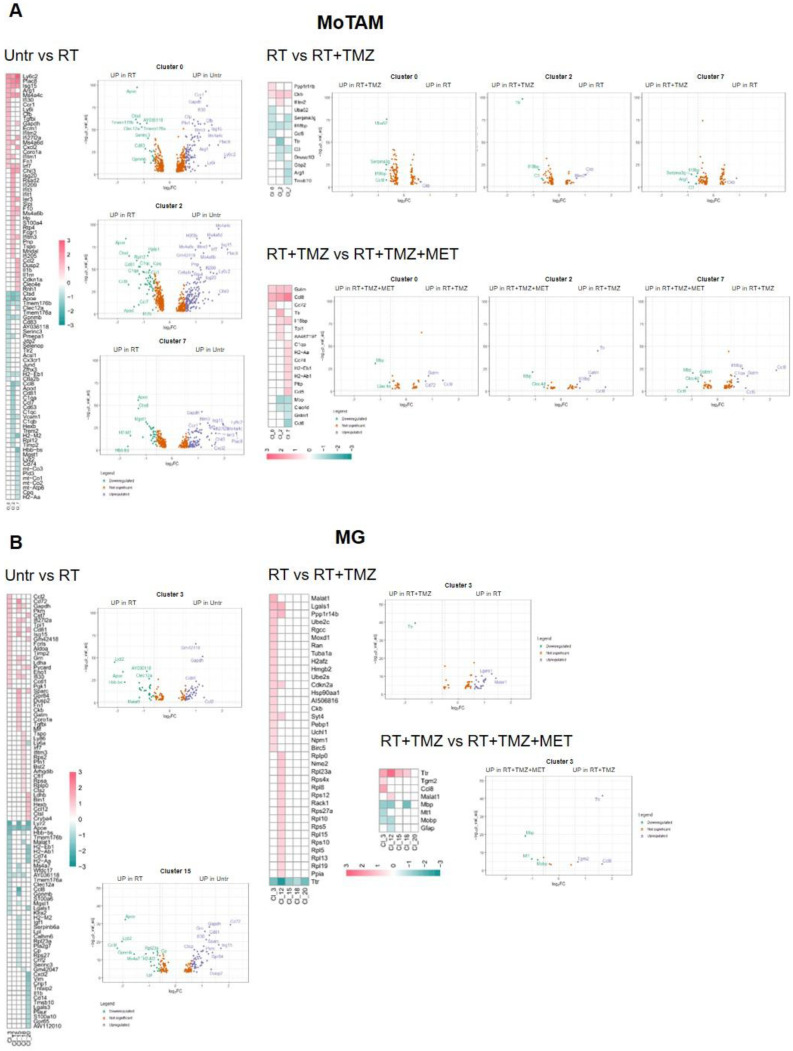
** RT and chemotherapy induced modifications in monocytes/tumor associated macrophages and microglia. A** Heatmap of the common differentially expressed genes analysis between untreated versus RT, RT versus RT+TMZ and RT+TMZ versus RT+TMZ+MET in monocytes/TAMs clusters and the corresponding volcano plots of CL 0, 2 and 7. MoTAM: monocytes/tumor associated macrophages. **B** Heatmap of the common differentially expressed genes analysis between untreated versus RT, RT versus RT+TMZ and RT+TMZ versus RT+TMZ+MET in microglia clusters and the corresponding volcano plots of CL 3 and 15. Up-regulated genes of the first term of comparison are highlighted in blue, down-regulated genes of the first term of comparison are highlighted in green, and not significant genes are highlighted in orange (Log_2_FC > ±0.3; Adj p<0.05).

**Figure 9:**
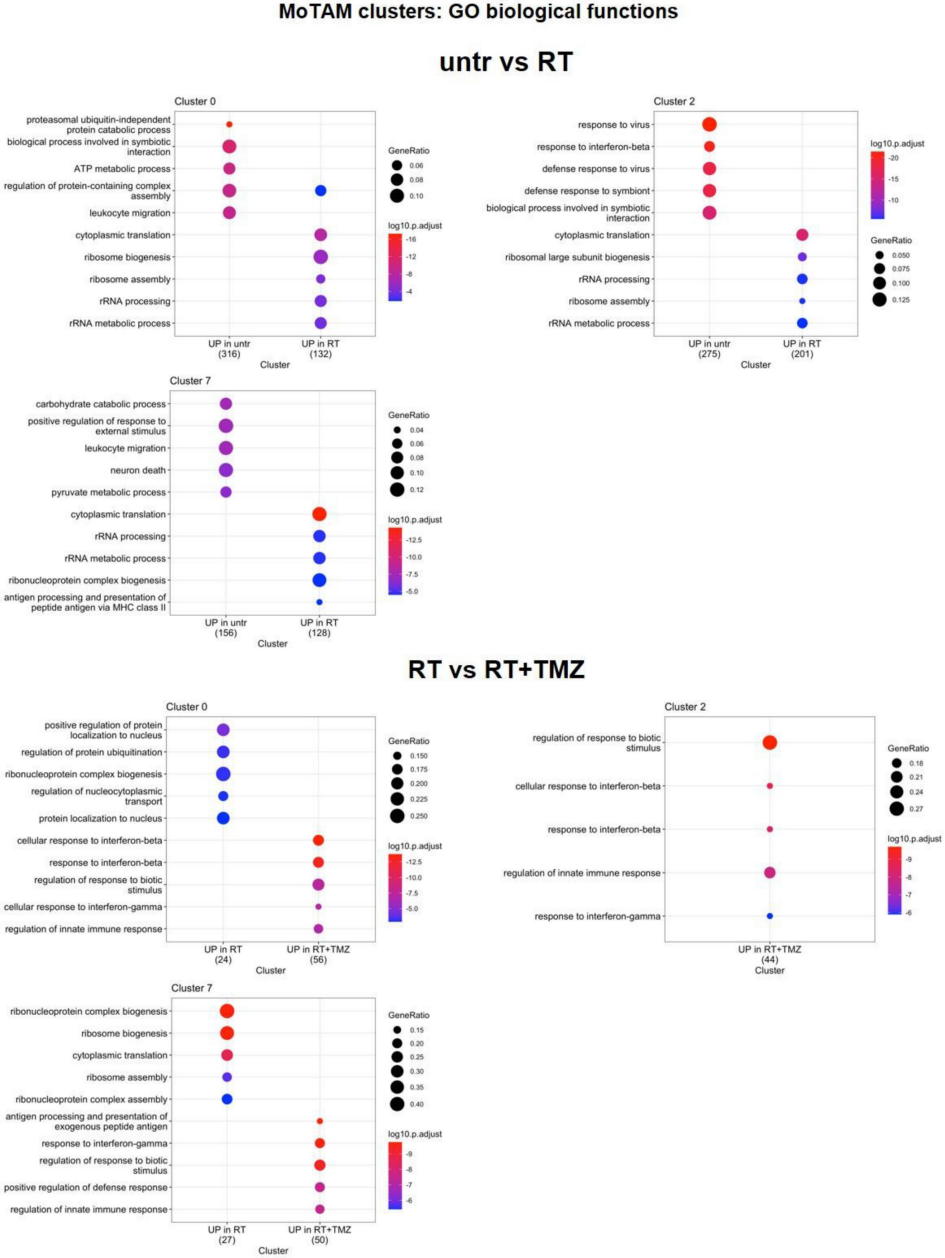
** Modification in biological functions after therapy in MoTAM clusters.** Top upregulated and downregulated biological functions in monocytes/tumor associated macrophages clusters 0, 2, and 7 obtained by gene ontology (GO) analysis of significant DEGs previously identified among the groups: untreated versus RT alone, RT alone versus RT plus TMZ.

**Figure 10:**
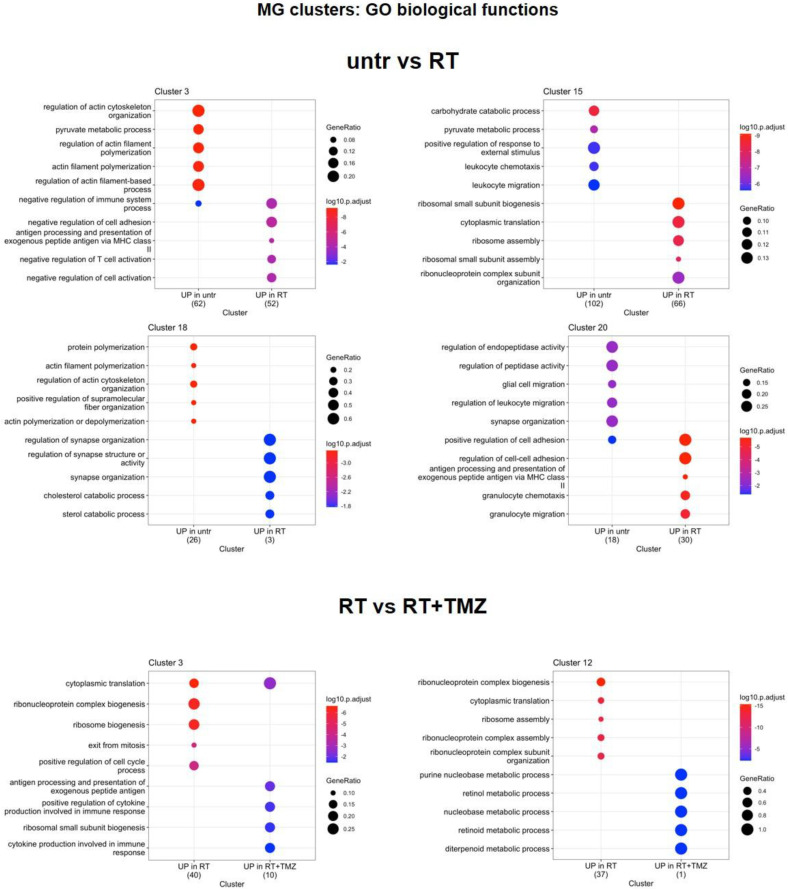
** Modification in biological functions after therapy in microglia clusters.** Top upregulated and downregulated biological functions in microglia clusters 3, 12, 15, 18, and 20 obtained by gene ontology (GO) analysis of significant DEGs previously identified among the groups: untreated versus RT alone and RT alone versus RT plus TMZ.

**Figure 11:**
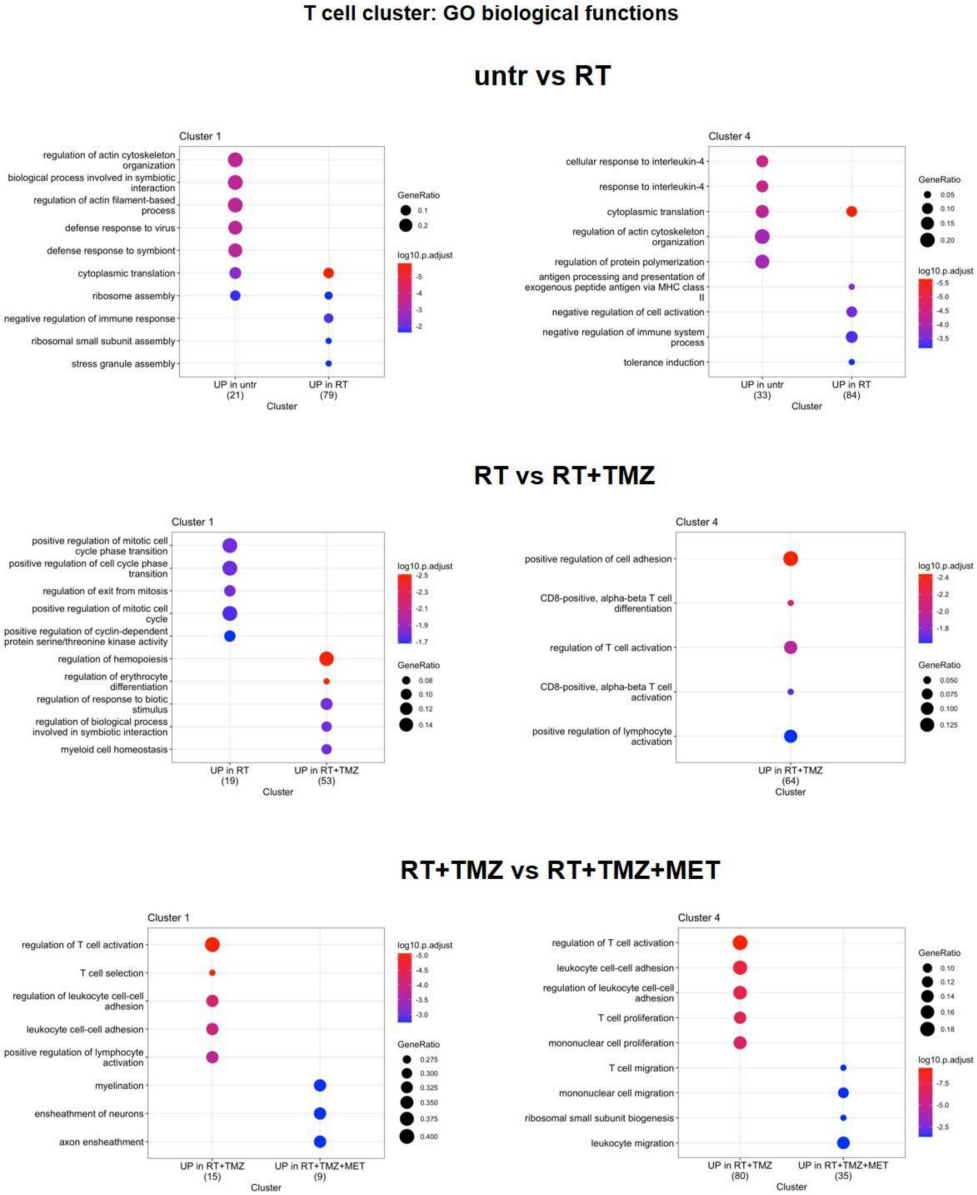
** Modification in biological functions after therapy in T cells clusters.** Top upregulated and downregulated biological functions in T cells clusters 1 and 4 obtained by gene ontology (GO) analysis of significant DEGs previously identified among the groups: untreated versus RT alone and RT alone versus RT plus TMZ.

**Figure 12:**
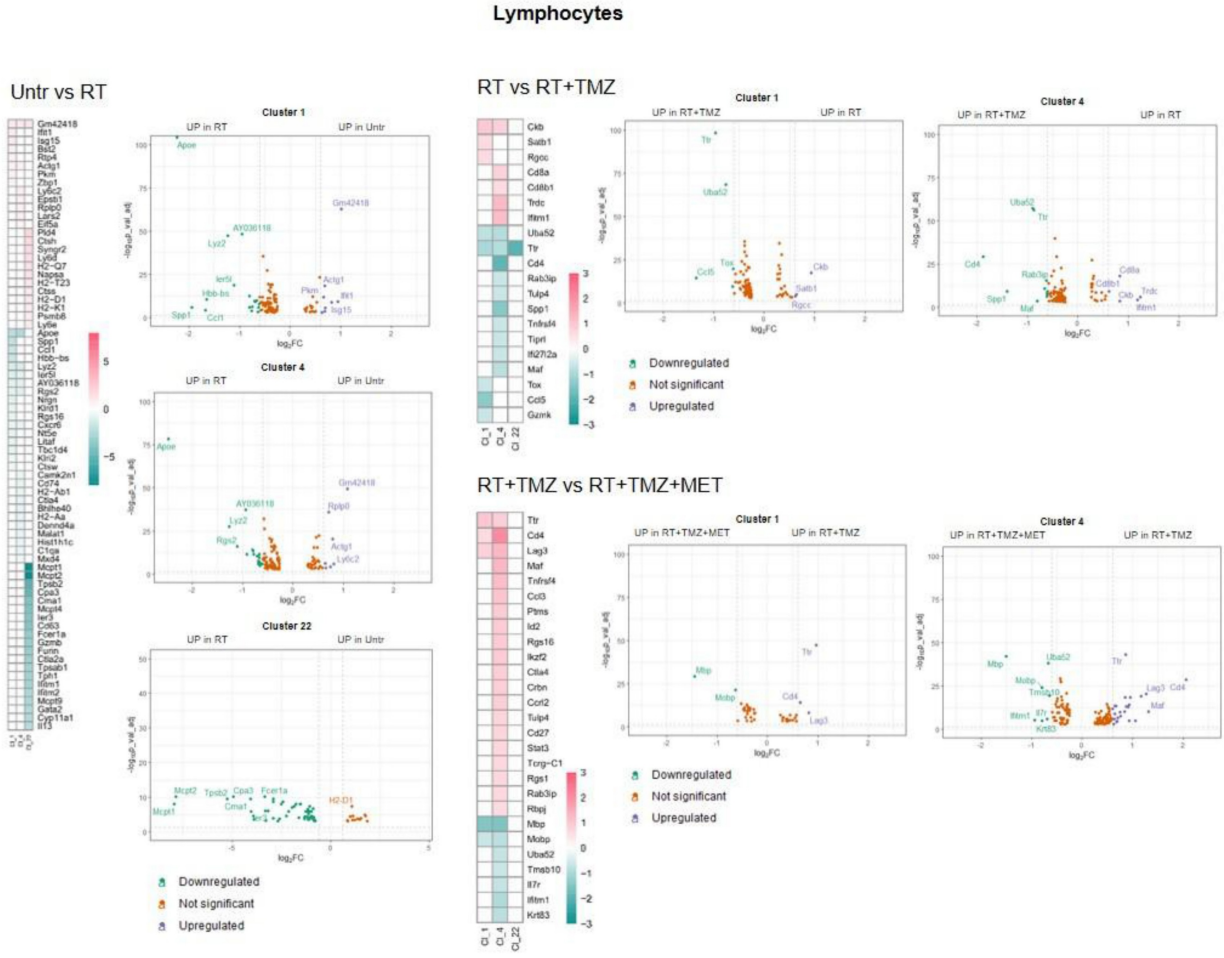
** RT and chemotherapy induced modifications in lymphocytes.** Heatmap of the common differentially expressed genes analysis between untreated versus RT, RT versus RT+TMZ and RT+TMZ versus RT+TMZ+MET in lymphocytes and the corresponding volcano plots of CL 1, 4 and 22. Up-regulated genes of the first term of comparison are highlighted in blue, down-regulated genes of the first term of comparison are highlighted in green, and not significant genes are highlighted in orange (Log_2_FC > ±0.3; Adj p<0.05).

As shown in Figure 8A, several TAM markers (*ApoE*, *Ctsd*, *Gpnmb*, and *Ccl8*) were upregulated in the majority of clusters after radiotherapy (RT). In contrast, *Ly6c2*, interferon pathway transcripts (*Isg15*, *Ifitm3*, *Irf7*), *Ms4a4c*, *Arg1*, and *Plac8* (involved in radiosensitivity) were downregulated, along with *Cxcl2*, *Ccr1*, *Il1b*, *Gapdh*, *Tgfbi*, and *Tspo*. Overall DGE analysis suggests a worsening of TAM phenotype particularly of Mo7 probably driven by CL9^66^. Also in Mo, RT increased annotation associated with *ribosomes synthesis and translation* while reducing pro-inflammatory immune functions and glycolytic metabolism. C*arbohydrate catabolic and pyruvate metabolism* were down regulated in MoTAM 7 and *ATP production and protein catabolic processes* in Mo0 (Figure 9, Supplementary Data 7 and 10). Association of TMZ increased the native immune response in all clusters while reducing ribosome annotations. However, an increase in some TAM (*Ccl8*, *Arg1, C3*) and to a minor extent immunosuppressive markers (*Cd274* and *Pdcd1lg2)* was also observed Supplementary data 10. A different signature was displayed in presence of MET with TAM transcripts (*Ccl8, Gatm* 67,68), chemotactic and immunosuppressive factors downregulated, particularly in Mo7 resembling a phenotype closer to early-stage tumor 31. As for Mo, *ApoE* and *Lyz2* were upregulated after RT in most of MGs. MG3 and MG20 increased MHC II transcripts and remodeling markers. TAM markers (*Ccl8, Gpnmb, Ms4a7*) 69 were upregulated in MG15, that down regulated immunosuppressive genes like *Cd274, Mif* and *Cd72* this last also in MG3 (Figure 8B, Supplementary Data 10). In MG3 and MG18, an increase of *Malat-1* was also observed. GO analysis (Figure 10, Supplementary data 7) showed a cluster specific modulation of MGs. Ribosome functions were among the top increased annotations only in MG15. In Mo7, MG3, and MG15, glycolysis was reduced, along with chemotaxis, immune response, and cytoskeleton organization. This last reduction, also observed in MG18, suggests a less reactive phenotype. On the contrary, cluster 20 upregulated cell adhesion, MHC II presentation, and chemotaxis while reducing regulation of peptidase activity and synaptic organization. TMZ add-on counteract RT induced modifications of immune reactivity only in MG3, increasing antigen presentation and cytokine production annotations while reducing *Malat-1* levels. Differently to what was observed for peripheral infiltrated macrophage, MET had minimal effects on microglial except for a reduction of *Malat-1* in MG12 (Figure 8B, Supplementary data 10). RT increased ribosome function also in lymphocytes, while reducing immune reactivity and metabolism particularly in Treg, an effect counteracted by TMZ (Figure 11, Supplementary data 7). Immunosuppressive genes 31 (*Ctla4*, *Cxcr6*, *Fasl* and *Cd279*) were increased after RT. This effect was blocked by TMZ-MET association, which showed a reduction in *Lag3, Maf, Ikzf2, Stat3, Ctla4* and to a minor extent *Cxcr6* and *Tox* in exhausted T cells CL4 and *Lag3, Cxcr6* and *Tox* in Treg*.* Overall MET add-on reduced the immunosuppressive phenotype of TME acting on both Treg and exhausted T cells, involving for this last the exhaustion driver *Maf* 70 and *Stat3* down regulation (Figure 11-12, Supplementary data 11). In line with the vascular effects, after RT, B cells increased functions associated with endothelial modulation, matrix remodeling and peripheral immune cells recruitment/activation (Figure 12 and Figure S10, supplementary data 7). Finally, the highest increase in ribosome functions was detected in NK cells. Overall, an immune response driven by residual tumor cells persisted at 4 weeks post-RT, characterized by a sustained increase in peripheral monocyte and Treg recruitment, metabolic and phenotypic modifications resembling TAMs, and a stress cell response, as indicated by elevated ribosome-related annotations. This effect was partially counteracted by TMZ, although an increase in Treg cells, along with immune evasion markers, was also observed. Notably, MET was able to reduce the immune-escaping milieu, particularly affecting lymphocytes and the relative abundance of Treg cells. Our results indicate how residual tumor cells influence both innate and adaptive immune reactions, favoring tumor progression. The combination of TMZ or TMZ plus MET enhanced immune system efficiency, with TMZ primarily impacting myeloid cells and MET primarily impacting lymphocytes.

## Discussion

This study evaluated the efficacy of the TMZ-MET combination in the GL261 syngeneic model, examining its molecular effects on the immune system. To elucidate tumor microenvironment (TME) modifications, a single-cell analysis-based experimental paradigm was employed, and in alignment with clinical practice, radiotherapy (RT) was incorporated into the *in vivo* experiments. RT-drug combination increased survival time in comparison to drugs or RT given alone, but differently to what observed in other mice models, the efficacy of TMZ and TMZ plus MET was similar. Bulk analysis performed ex-vivo, showed a reduction of G1/S cell cycle markers immediately after RT but this effect, particularly evident for *CycD1* was maintained over time only in presence of drugs.

Single-cells analysis explained the large variability observed from PCR analysis, confirming the effects on cell cycle. In agreement with genomic analysis, tumor region was heterogeneous showing four different clusters with distinct signatures influencing treatment sensitivity. All clusters showed lower response to RT compared to RT-drug combination. Notably, CL9 displayed the highest radioresistance but conversely, the greatest sensitivity to the drug combination. Four weeks post-RT, we observed increased ribosomal function and cytoplasmic translation annotations in all clusters, except for the radioresistant CL9. The arrest of translational processes, is a hallmark of cellular stress response and serves to minimize energy expenditure and restore homeostasis following external insult 71. Gao et al.^59^, reported an early increase in ribosomal function post-RT, revealing heterogeneous cellular responses, with radioresistant clusters showing minimal changes in ribosome-associated genes. We observed persistent upregulation of translational functions in the cancer sub clusters more sensitive to radiations.

Conversely, in line with what observed by Gao et al.^59^, the less responsive CL9 showed reduced translational and ribosome functions alongside elevated levels of *Npm1,* a protein transcript involved in DNA repair. In CL5 and CL8, translational apparatus modifications were accompanied by increased mitochondrial function annotations, suggesting metabolic adaptation to the elevated energy demands of restoring protein synthesis and mitigating oxidative stress. Indeed, in response to stress vulnerable cells induced pro-survival mechanisms including stimulation of metabolic activity 72. Conversely, CL9 maintained a glycolytic phenotype, concurrently increasing inflammatory functions and influencing TME composition 73-76. A similar stress response was observed in a subset of immune cells, which exhibited increased ribosome biogenesis and *Apoe* levels, an effect already observed in other cancer model 77,78.

Another relevant point for RT resistance was the general increase of *Malat1.* Post RT, *Malat1* was overexpressed in all residual cells and CL9 exhibited the highest differential expression. *Malat1* overexpression is linked to tumor progression and chemo resistance including TMZ 57. Emerging evidences suggest its involvement also in radio-resistance. *Malat1* silencing significantly enhances the radio sensitivity in malignancies like nasopharyngeal, lung and cervical cancer modulating miRNA and pathways affecting DNA repair, cell cycle, apoptosis, stemness and TME reprogramming towards immunosuppression (See 79 for a review). Furthermore, elevated *Malat1* levels have been reported in radiotherapy-resistant oncologic patients 80. Consistent with these findings, we propose that the post radiation increase in *Malat1* transcript in our study, contributes to the low radio- sensitivity of residual cells and the enhanced immune evasive TME 81. As expected GL261 model showed an important inflammatory response characterized by the presence of immunosuppressive cell clusters 40. In line with IF and [^18^F]VC701 PET, single-cell analysis revealed a complex and heterogeneous milieu that included myeloid, lymphoid and parenchymal cells influenced by the tumor with resident and infiltrating monocytes representing the highest fraction 28,33,40. Manual annotation of DEGs showed ligand-receptors pairs transcripts similar to those described in another glioma model 48, suggestive of a complex interaction between tumor-TME and treatment 82. Immediately after the administration, RT reduced intra-tumor microglia, an effect maintained over time only in presence of drugs. This reduction was limited to TMEM119/IBA-1-negative cells, suggesting rapid remodeling of a microglial subpopulation implicated in neurogenesis and synaptic repair, according to some authors 83. Resident and peripheral infiltrating monocytes showed a reduced glycolytic phenotype and in line with the metabolic modifications 84 of inflammatory reactivity. Peripheral originating CL7 displayed a comparable response to irradiation demonstrating a worsening of TAM phenotype. These effects were not observed in the highly proliferative MG20, whose levels decreased in the presence of drug association, presumably positively involved in tumor control, or in MG18, which carried the highest levels of homeostatic transcript, thus resembling recently recruited cells. In MG3, MDSCs and peripheral originating TAM, inflammatory responses were partially restored by TMZ. However, TMZ was not able to counteract the immunosuppressive milieu still present despite the reduced proportion of tumor cells 48. The addition of MET showed no effects on MG but modified myeloid cell transcripts towards phenotypes resembling those in low-grade tumors or normal tissue, with a slight increase in MHC II and a reduction in those involved in immune evasion, angiogenesis, and tissue remodeling. *Malat1* was also modulated in MG clusters, in a treatment-dependent manner*.* As observed for tumor, RT increased the lncRNA, an effect counteracted by TMZ or TMZ-MET suggesting a role of MALAT-1 to sustain the pro-tumoral signature of microglia, a phenomenon previously described for tumor associated macrophages in breast cancer 85. RT reduced immune response also in DCs and lymphocytes clusters, increasing Treg, and immunosuppressive transcripts in both CD4+ and CD8+ cells and inducing a less responsive and indolent phenotype 86. Drug treatments reduced CD3 positive cells. However, TMZ was not able to modify the immuno-evasive phenotype while maintaining Treg cells representation and remodeling cluster 4. Mice treated with TMZ showed an enhanced vascularization of tumor indicated by an increase of CD31 marker and endothelium cluster representation. Remarkably, MET add-on inhibited these effects, reducing the expression of *Lag3* and other immunosuppressive markers (*Maf, Cxcr6, Ikzf2, Ctla4, Tox, and Stat3*) 31 in Treg or CD8+ T-cells. Notably, inducers such as *Tox*, which we found downregulated in the MET add-on group 87, regulate *Lag3* transcription. The effects of MET on T-cell reactivity have been previously described in other cancers. In murine models, MET restored CD8+ T-cell reactivity *in vitro* and *in vivo*, attenuating the upregulation of LAG3 and PD1. MET administration synergized with cyclophosphamide, enhancing the response to adoptive immune therapy or checkpoint blockers 88. In pancreatic ductal adenocarcinoma (PDAC), MET reduced LAG3 and STAT3 levels, and increased overall survival 89. The molecular mechanism by which MET modulates T cells is not fully understood, with both AMPK-dependent and -independent mechanisms proposed 87,90,91. Overall, our results in GBM confirm the modulatory effect of MET on immune-depressive factors, such as LAG3 and STAT3 lymphocytes functions and endothelium, as previously described in other tumors 88,92.

The syngeneic model used in our study shows several genetic similarities with human signature, and has the advantage to include T cells response with a general good adherence with glioma patients TME 28,40. However, it cannot fully recapitulate the intra- and inter-tumor heterogeneity of GBM 22. For this reason, despite the novelty of these findings, the effect of MET as a potential immunomodulator in glioma warrants further confirmation in other mouse models and in patients, including measuring these markers at the protein level 87. This is even critical because the role of LAG3 in glioma and the potential benefit of its modulation is not well established yet 93,94.

This study confirms the potential of [^18^F]FLT as a predictive diagnostic tool for radio-chemotherapy response. Currently, PET, associated with amino acid radiopharmaceuticals, is used for the delineation of tumor margins and recurrence 95. While “Stupp” protocol is the unique standard therapeutic intervention, limiting the immediate clinical relevance of response prediction, it holds significant interest for developing novel cell-cycle modifier treatments. However, results of our study indicate that *Tk1*, the substrate of [^18^F]FLT, is present also in immune cells and not uniformly distributed in tumor. These factors should be carefully considered during [^18^F]FLT PET application and interpretation. Differently to what previous observed, in this model PET with [^18^F]VC701 was not associated with response. From single cells analysis, we observed a non-exclusive but prevalent association of its target (TSPO) with dendritic cells, particularly mDC subtype, microglia sub-clusters and the less mature infiltrating monocytes with levels of expression poorly modulated by treatment. Overall, these results suggest that [^18^F]VC701 PET signal may solely reflect changes in overall inflammation without providing information on phenotypes. Thus, its predictive use should be limited to treatment where the reduction of the inflammatory component of tumor and not its phenotype is part of the response. Despite the highest effect on cell growth shown *in vitro*, MET add-on failed to prolong GL261 mice survival. As previously mentioned, MET inhibits complex I of the mitochondrial electron transport chain, reducing NADH oxidation and tricarboxylic acid (TCA) flux 4. Cancer cells unable to compensate for metabolic stress undergo apoptosis 96. Conversely, cells with rewired metabolic pathways are less sensitive to MET effects. In residual tumor cells, post RT, mitochondrial respiration and glycolysis modification were unaffected by TMZ or TMZ+ MET administration. GL261 cells harbor mutations in *Kras* and *Trp53* 22, *Pten* deletion, and *Myc* amplification, a genetic signature driving metabolic reprogramming and reducing MET effects 97-99. However, distinct studies showed that MET can block cancer growth independently of p53 activity 100, inhibit metabolic rewiring in both Kras mutant and PTEN-deleted models 101,102, and reduce MYC levels 103. These effects, which have not been described in glioma, warrant further evaluation regarding MET efficacy.

## Conclusion

In this study, we showed that tumor cells residual to RT exhibited a heterogeneous strategy to counteract radiation damage, involving ribosome apparatus activation, metabolic rewiring, and immune response, promoting a pro-tumoral inflammatory milieu. This last strategy was common also to subset of cells surviving to radio-chemotherapy combination, driving the inflammatory milieu still highly represented during chemotherapy although with a more reactive phenotype. Off note, MET add-on counteracted the immuno-evasive profile of GL261 model involving particularly T cells suggesting a potential role of MET also in adopted immunity. Finally, modulation of MALAT-1 stresses the relevance of a better understanding of the role of lncRNAs and related miRNAs in GBM radio-chemotherapy resistance 104,105.

## Materials and Methods

### Cell Culture

Murine GBM GL261 cells (purchased by DSMZ [https://www.dsmz.de/collection/catalogue/details/culture/ACC-802]) were used for research. GL261 cell line, carrying mutations in TP53 and KRAS genes, derives by 3-methylcholanthrene-mediated induction of tumors in C57BL/6 animal models and their maintenance through serial transplantation in a syngeneic mouse model 22. Cells were cultured in high glucose Dulbecco's Modified Eagle Medium (DMEM) (Euroclone), supplemented with heat-inactivated Foetal Bovine Serum (FBS) (10%), 50 IU/ml Penicillin/Streptomycin (P/S), 2 mM glutamine (all Euroclone, UK) at 37°C in a 5% CO_2_ and 95% air atmosphere.

### Cell Treatments

Five x 10^4^ cells/well were plated in 24 multi wells, and after 24 hours exposed to increasing doses of TMZ (10, 25, 100 µM) or MET (1, 5, 10 mM) (both Sigma Aldrich, St. Louis, MO, USA); cell growth was monitored for 24 and 48 hours. Cell viability assay was performed by Trypan blue exclusion test. Based on the results obtained (data not shown) 5 mM MET was chosen and added to 25 or 100 µM TMZ for 24 and 48 hours and cell viability evaluated. The effect of MET or TMZ was determined as growth inhibition rate and measured as: [1-(Cf/C0)A/(Cf/C0)V]*100, where Cf is the cell number at the point analyzed, C0 is the cell number at the beginning of treatment, A is the corresponding drug and V is the vehicle as previously described 7. MTT assay (Sigma-Aldrich, Merck) was performed to analyze treatment toxicity at the same conditions used for MET and TMZ combination study at 24, 48 and 72 hours. Additional 150,000 cells/well were plated in 6 multi wells, and after 24 hours exposed to 25, 100 µM TMZ given alone or in combination with 5 mM MET for 24 hours, collected and lysed for the Trizol-mediated RNA extraction.

### GL261 genomic analysis by array CGH

Genomic DNA was extracted by Maxwell® Promega instrument (Promega, Milan, Italy). Sample preparation, slide hybridization and analysis were performed using Mouse 2x150K Microarray HD-CGH (Agilent Technologies, Santa Clara, CA, USA), according to the manufacturer's instructions. Mouse Genomic DNA commercial sample from mouse blood (Promega, Milan, Italy) was used as reference DNA during array-CGH. The arrays were scanned at 5-µm resolution using Agilent microarray scanner and analyzed using Feature Extraction v10.7 and Agilent Genomic Workbench v7.0 software. The Aberration Detection Method 2 (ADM2) algorithm prompted by Genomic Workbench software was used to compute and assist the identification of aberrations for a given sample (threshold = 5; log2 ratio = 0.3). To calculate the estimated percentage of mosaicism we used the formula determined by Cheung SW et al. 106. All nucleotide positions were referred to the Assembly NCBI37/mm9 (July 2007) Reference Sequence. MGI Mouse Genome Informatics (informatics.jax.org) database was used for Gene Ontology (GO) and homology information.

### Cell migration and invasion: wound healing and Boyden's chamber

To study directional cell migration by wound-healing assay, the GL261 cells were seeded at a confluence of 150,000/well in a 24-wells plate (Euroclone, UK). After 48 hours, necessary to reach 80% confluence, a scratch was performed in the middle of each well. After washing, the medium was replaced, and the pharmacological treatments were applied, as described before. Pictures of each well were taken every 24 h. The effect on cell migration was quantified by ImageJ software. The quantification of the wound was performed by calculating the area of the scratch at 24 hours normalized on the area in the same point of the well at time 0. This means that if a treatment reduced the capability of the cells to restore and fill up the wound, the ratio of the area 24 hours/0 hour should increase compared to untreated or scramble treated cells, following the method described by Grada and colleagues 107. Experiments were performed in triplicate. A standard Boyden's chamber test was used for the invasion study. The test was performed and quantified as described previously 108.

### RNA Extraction and Real-Time PCR on cells

RNA was extracted by the Trizol method, following the manufacturer's instructions. Total RNA was reverse transcribed to cDNA using the High Capacity cDNA Reverse Transcription Kit (Thermo Fisher Scientifics, USA). Real-time PCR was performed in duplicate for each data point by using the Sybr Green technique (SsoAdvanced Universal Sybr Green Supermix by BioRad) and the oligonucleotides used were: 14S, TK1, CycD1, CDK2, CycA2, CDK1, PKM2, HK2 Supplementary Table 8. Changes in the target mRNA content relative to housekeeping (14S) were determined by calculating the fold change expression (as the 2^-ΔΔCT^± sd)^109^. The analyses were performed in triplicate for vehicle and RT conditions and duplicate for RT+drugs.

### Animal Models and Treatments

Animal study was reviewed and approved by the Ethics Committee of IRCCS San Raffaele Scientific Institute of Milan and Italian Ministry of Health (n. 378/2019-PR). Seven to eight weeks old female C57BL/6 mice (Envigo RMS, San Pietro al Natisone, Italy), were housed at constant temperature (23°C) and relative humidity (40%) under a regular light/dark schedule. Food and water were available *ad libitum*. The orthotopic tumor model was obtained by the stereotactic injection of 1x10^5^ murine GBM GL261 cells (ACC 802, Leibniz Institute DSMZ, Germany) in 2 µl of plain DMEM with a 10 µl Hamilton syringe as previously described 9. After cell injection, mice were monitored every day for body weight and clinical signs of disease (fur, eye, motor impairment) and sacrificed at the appearance of evident signs of illness or loss of more than 25% of the initial body weight. In a first group of mice, the effect of vehicle, MET (D150959, Merck), TMZ (T2577, Merck) or TMZ plus MET on tumor growth was evaluated. Tumor bearing mice were randomly assigned to 4 groups of treatment, according to the following scheme: group A received daily oral administration of TMZ (70 mg/kg) in 10% DMSO, 5 days for a 28 days cycle and repeated with this scheme (5/28) until sacrifice of animal; group B received daily intra peritoneal (i.p.) administration of MET (250 mg/kg) in saline for 5d/wk for the entire treatment period; group C received the combination of daily oral administration of TMZ (70 mg/kg) 5 days for a 28 days cycle and i.p. daily administration of MET (250 mg/kg); group D, as vehicle group, received vehicle (10% DMSO in saline by oral gavage and 100% saline i.p.). The treatment schedule was decided on the basis of previous studies 9 and of the dose regimen used in clinical practice adapted to mice body surface 9. The effect of radiotherapy given alone or with drugs was subsequently evaluated in additional groups of mice (vehicle n=10; RT n=6, MET n=4, TMZ n=6, TMZ+MET n=5, RT+TMZ n=7, RT+TMZ+MET n=5). Radiation therapy (RT) was administered in three consecutive daily fractions of 8 Gy each, for a total dose of 24 Gy. This hypofractionated regimen was selected based on a previous study 110 demonstrating comparable efficacy between a hyperfractionated regimen (16 x 2 Gy) and the current hypofractionated regimen (3 x 8 Gy). The hypofractionated approach offered the advantage of reducing the number of anesthetic exposures required for the mice, while both regimens yielded similar effects on the immune system.

Radiotherapy was performed as follows: before receiving the radiation treatment, animals were anesthetized with gaseous anesthesia (2-3% isoflurane and 1 l/min oxygen) and positioned prone on the animal bed of small animal dedicated rotating (360°) irradiator equipped with cone beam computed tomography (CBCT) guidance (X-RAD225Cx SmART, PXI North Branford, CT, USA). Tumors were contoured on the CBCT images and two equal-sized dose beams were set at 90° and 270° respectively. Tumor dose distribution was calculated by means of a Monte Carlo based treatment planning system (SmART-ATP) and the mean dose adjusted to 8 Gy per fraction. The irradiation settings were: tube voltage = 225 kVp, current = 13 mA. Delivery time was about 90 seconds/field with a total duration of the procedure of 20 min. After the treatment, animals were replaced in their cages until the complete awakening and treated with vehicles or drugs. Drug treatment (TMZ, TMZ plus MET or Vehicle) began at the end of the first day of RT administration, and mouse health was monitored as previously described.

For efficacy studies, mice were sacrificed based on clinical signs as previously described with a maximum frame of 4 cycles of TMZ. The two treatment schedules (presence or absence of RT) were pooled and treatment efficacy assessed as time to sacrifice indicated as “overall survival” using the Kaplan-Meier method. Post treatment PET imaging with [^18^F]FLT and [^18^F]VC701 and post mortem molecular analysis were performed only in the RT, RT plus drugs groups because of COVID-19 emergency. Number of animals per condition and experiments are summarized in Supplementary Table 9. Imaging studies and ex-vivo analysis are detailed below.

### *In vivo* Imaging

All experiments were performed on a 7-Tesla MRI scanner (Bruker, BioSpec 70/30 USR, Paravision 6.0.1, Germany) fully equipped for *in vivo* imaging in mice.

Mice were anesthetized using 1.5-2% isoflurane vaporized in 100% oxygen (flow rate: 1 L/min) and placed on a warmed bed to maintain a body temperature of 37°C. Respiratory rate was continuously monitored to adjust anesthesia throughout the MRI examination. A mouse brain surface array coil (receiver) was positioned over the animal's head, with a volume coil acting as the transmitter. Coronal T2-weighted images, used for tumor volume quantification, were acquired across the entire brain using a 2D-TurboRARE sequence with the following parameters: field of view (FOV) 1.9 × 1.4 cm, matrix size 170 × 170, 18 slices with 0.75 mm thickness, spatial resolution 0.082 × 0.12 mm, repetition time (TR) 3000 ms, echo time (TE) 48 ms, RARE factor 10, and 5 averages. Tumor volume was calculated through MIPAV software by manual contouring the T2 sequences acquired. MRI scans were performed before the beginning of treatment. In RT-drugs combination groups, MRI was performed also at 2 and 4 weeks after the beginning of therapy and then monthly until the end of the study. In the same group of mice, PET scans were carried out at 2 and 4 weeks after treatment beginning with [^18^F]FLT and [^18^F]VC701 to determine tumor proliferation and inflammation respectively 9. Briefly, mice were intravenously injected with 3.848 ± 0.247 MBq of [^18^F]FLT or with 4.585 ± 0.591 MBq of [^18^F]VC701. PET acquisitions were carried out with a small animal dedicated PET-CT scanner (X-ß-CUBE, Molecubes, Gent, Belgium). After calibration and correction for physical half-life of [^18^F]fluorine, PET/CT images were co-registered to MRI scans and analyzed using PMOD 4.105 software (Zurich, Switzerland). A first volume of interest (VOI) was drawn on contralateral normal brain parenchyma (CL) at the levels of the left corpus striatum and a second centering tumor margins. Both VOI were defined on axial MRI and then copied on [^18^F]VC701 and [^18^F]FLT PET images. Radioactivity concentration values were expressed as Standardized Uptake Value (SUV) using the following formula: radioactivity concentration in tissue (decay corrected MBq/cc) divided for the injected doses (decay corrected MBq/cc) multiplied for animal weight (gr). SUV values were calculated as SUV max for tumor and SUV mean for CL to obtain tumor to CL (background) ratios values (T/B). PET images were not performed in the first cohort of mice because of lack of access to imaging systems during COVID-19 emergency.

### Immunofluorescence analyses

Mice brains were collected at sacrifice and fixed in paraformaldehyde solution 4% in PBS as described 111. After the standard brain inclusion process, serial 18 µm-thick brain sections were cut and stained with different immune response-related markers (CD16, CD206, TMEM119, Iba1, CD3, TSPO and GFAP). After washing the slices with PBS, slides were incubated in 1X Reveal Declocker (1-800-799-9499 BioCare Medical, Pacheco CA, USA) for 20 min at 95 °C (antigen retrieval), then incubated with Glycine 0.3% 20 min RT. After 3 washing, slides were incubated with blocking buffer (1% BSA, Donkey serum 5% and TritonX-100 0.3% in PBS) 45 min at RT and then either a primary antibody diluted in blocking buffer (see Supplemental Table 8 for specifics of the primary antibodies) or just blocking buffer to serve as a negative control to exclude nonspecific staining by the secondary antibodies or channel bleed through. Following primary antibody treatment, slides were washed, then incubated for 1 h at room temperature with 1:500 dilution of species appropriate Alexa Fluor 488/568 labeled secondary antibody (Thermofisher Scientific, Waltham, MA, USA) in PBS. Because most of these primary antibodies were raised in the same species, co-localization of two proteins (Iba1, GFAP, TMEM, CD206 and TSPO) was performed using a nanobody staining procedure as previously described 112. Briefly, after first antibody staining the slices were washed 3 times for 5 minutes each with PBS and post fixed with 4% PFA for 10 minutes. In the meantime, the primary antibodies were premixed for 30 minutes with two molar excess of fluorescently-labeled Secondary Nanobodies (NanoTag Biotechnologies, Cat. No.: N1202-At565) in PBS. The pre-mixed complexes were then incubated on the fixed slice 4h, RT.

The co-localized samples were compared with samples where each primary antibody, as well as the non-binding blocking antibodies, were separately stained to ensure that the blocking was complete. Once fluorescently stained and coverslipped slides are obtained by these methods, they are imaged using DeltaVision Ultra system (GE Healthcare, Chicago, USA) equipped with a 20X/NA0.5 objective lens (Olympus, Tokyo, Japan). For multi-colour imaging, z-stacks of individual channels were sequentially acquired, after optimization of imaging parameters such as illumination parameters and exposure time. Consecutive images with 10% overlap were collected with Tile Scan. Tile Scan allows multiple images spanning the entire specimen with 10% overlap to be collected, and then computationally stitched as tile mosaic images using the grid/collection stitching plugin provided by the software package SoftWoRx provided by the microscope's manufacturer. The segmentation and analysis were performed using the software ArivisVision4D (ZeissAG, Germany). The segments of the specific areas (“tumor”, “peri” and “lateral”) on the entire tissue were created manually, and the area covered was measured. While the specific antigen positive staining areas were identified on the correspondent channel with a Local Adaptive Threshold algorithm. The sum of antigen object areas and the total tissue area were exported to calculate % of coverage for statistical analyses. Note that all samples were segmented with the same parameters in order to make the results comparable. To quantify CD3 staining, the number of cells (CD3 positive objects) was measured for each specific area and exported to calculate cell's density (number of positive cells/area). To identify TSPO staining the individual nuclei were identified with Blobs algorithm. Each nucleus has a unique identifier and the mask associated with the nuclei was opened of 5 pixels to detect Mean Intensity of TSPO staining at least in part of the cytoplasm. The mean intensity of all positive nuclei in “tumor” area was then normalized to the mean of the value in the “lateral” area. Note that all samples were segmented with the same parameters to make the results comparable.

### Tissue dissociation and Single-cell RNA sequencing and analysis

Immediately after sacrifice, GBM tumor samples of mice treated with vehicle, RT, RT plus TMZ, RT plus TMZ and MET were collected and dissociated enzymatically to obtain a single-cell suspension with the Tumor Dissociation Kit - mouse (130-096-730, Miltenyi Biotech, Bergisch Gladbach, Germany) and GentleMACS Octo Dissociator (Miltenyi Biotec), according to the manufacturer's protocol. Directly after tissue dissociation, cell concentration and viability were assessed. Single cells were processed for transcriptome analyses using the Chromium Controller (10x Genomics) and the *Chromium* Single Cell 3' Reagent Kits User Guide (v3.1 Chemistry) (10x Genomics) with a cell recovery target of 4000 single cells. Libraries were prepared following manufacturer's directions. After quality controls, libraries were sequenced on Novaseq platform (Illumina) aiming at 50,000 reads/cell. CellRanger v6.0.2 software (10X Genomics) was used to perform the preliminary steps of the analysis, including demultiplexing of the input FASTQ files, alignment to the mm10 mouse reference genome (Ensembl 98), UMI quantification and assignment to the GENCODE (v32) gene annotation. Cell-by-gene count tables for each sample were imported into R and analyzed with Seurat (v4.3.0) 113. Cells with lower than 700 UMI counts were filtered out from each sample (see Supplementary table 10), and the resulting matrices were log-normalized with a scale factor of 1000 by using the *NormalizedData* function. In each sample the 4,000 most variable genes were identified with the “vst” selection method of the *FindVariableFeatures* function. To remove possible batch effects among conditions, 4,000 anchor features were identified using the *FindIntegrationAnchors* method that exploited 20 Canonical Correlation Analysis (CCA) dimensions. Following this, samples were then combined into a unique object with the *IntegrateData* function. After quality filtering Figure S6, transcriptomes from approximately 21,000 cells were included in the analysis, with samples from the same condition pooled together (See Table S10 for cell numbers from each condition and mouse). Using the resulting batch-corrected embedding matrix, the Uniform Manifold Approximation and Projection (UMAP) dimensional reduction technique was computed to obtain 2-dimensions visualization of the cells. Clusters were identified using the original Louvain algorithm through the function *FindClusters* at the resolution of 0.8. To discriminate between mouse endogenous cells and orthotopic tumor cells, we took advantage of the *Souporcell* suite 114. Briefly, this method: remaps raw reads on the reference genome using *minimap2* (to avoid the false-positive variants introduced by the STAR aligner, which is part of the *cellranger* pipeline), calls candidate variants using *freebayes*, performs cell allele counting using *vartrix*, clusters the cells by genotype, and calls the doublets (cells with mixed genotype). To maximize the probability of identifying variants on mouse endogenous and orthotopic genotypes, candidate variants were extracted on the vehicle sample M2_veh, where tumor cells were expected to be abundant. Following this step, cells with mixed genotypes (9.6%) were removed. Additionally, we removed a small fraction of cells that were clustered by Seurat with cells of the opposed genotype (1.4%). Dimensionality reduction and clustering were performed again as described before on the cleaned dataset Figure S6D). To identify cell types present in our dataset, cluster markers were computed by using the *FindAllMarkers* function of the Seurat package. In detail, each cluster was compared to all the other cells by using a Wilcoxon Rank Sum test for significance. Genes expressed by a fraction of cells higher than 10% in either of the two groups of cells and having an adjusted p-value lower than 0.05 and a log2FC greater than 0.3 were considered markers. Moreover, to facilitate the identification of cell types, per-cell gene signatures were extracted using *CelliD* (115 and compared with a reference database of markers associated with well-established cell types (i.e., PanglaoDB; https://panglaodb.se/markers/PanglaoDB_markers_27_Mar_2020.tsv.gz]). Specifically, the *RunCellHGT* functionality was executed with 50 Multiple Correspondence Analysis (MCA) dimensions to calculate the top genes for the hypergeometric test. The assignment of cells to identities was based on the lower Benjamini-Hochberg adjusted p-value and cells exhibiting non-significant scores for any cell type were designated as unassigned. DGE analysis was performed using the *FindMarkers* function (with the same parameters described above) among the overall datasets and/or within each cluster to compare RT vs. untreated, RT +TMZ vs. RT, and RT+TMZ+MET vs. RT+TMZ. Significant DEGs among treatments were subjected to gene ontology (GO) analysis using the clusterProfiler package 116. Finally, volcano plots were generated using RStudio, Version 4.3.1. (2023-06-16 UCRT). Significant differentially expressed genes (DEGs) were selected based on their adjusted p value ≤ 0.05 and log2FC > 0.3 or <-0.3 among the groups. Top 20 upregulated and downregulated DEGs were selected for the creation of the Heatmaps using RStudio; when there were less than 20 genes all genes were used.

### Statistical Analysis

Experimental results are reported as mean values ± SD. The statistical analysis used is detailed in each figure legend. For statistical analysis we used GraphPad 9.5.1 Software Inc., CA, USA. Treatment efficacy was evaluated as time to sacrifice indicated as “overall survival” using the Kaplan-Meier estimator. Log-rank Mantel-Cox test was performed for survival comparison. ROC analysis of [^18^F]FLT Tmax/B for prediction of different response to radio-chemotherapy was performed using GraphPad 9.5.1 Software.

## Supplementary Material

Supplementary figures and tables.

Supplementary data 1.

Supplementary data 2.

Supplementary data 3.

Supplementary data 4.

Supplementary data 5.

Supplementary data 6.

Supplementary data 7.

Supplementary data 8.

Supplementary data 9.

Supplementary data 10.

Supplementary data 11.

Supplementary data 12.

Supplementary data 13.

## Abbreviations

DC: dendritic cell
DEGs: differentially expressed genes
DGE: differential gene expression
GBM: glioblastoma
[^18^F]FLT: 3′-deoxy-3′-[^18^F]fluorothymidine
lncRNAs: long non-coding RNAs
MDSCs: myeloid derived suppressor cells
MET: metformin
MG: microglia
Mo: infiltrating monocytes
NK: natural killer
PET: positron emission tomography
RT: radiotherapy
scRNA-seq: single-cell RNA sequencing
SUV: standardized uptake value
TAM: tumor-associated macrophages
TK1: tymidine kinase 1
TME: tumor microenvironment
TMZ: temozolomide
TSPO: 18 kDa translocator protein
