# Supplementary material for: Radio-chemotherapy and metformin selectively modulate the heterogeneous landscape of glioma with ribosome biogenesis, long non coding RNA and immune-escape markers as major player
Source: Int J Biol Sci. 2025 May 27;21(8):3527–54. doi: 10.7150/ijbs.103194 (PMC12160513; doi:10.7150/ijbs.103194)
Supplement: Supplementary file 1 — Supplementary figures and tables. [file ijbsv21p3527s1.pdf]

Figure S1

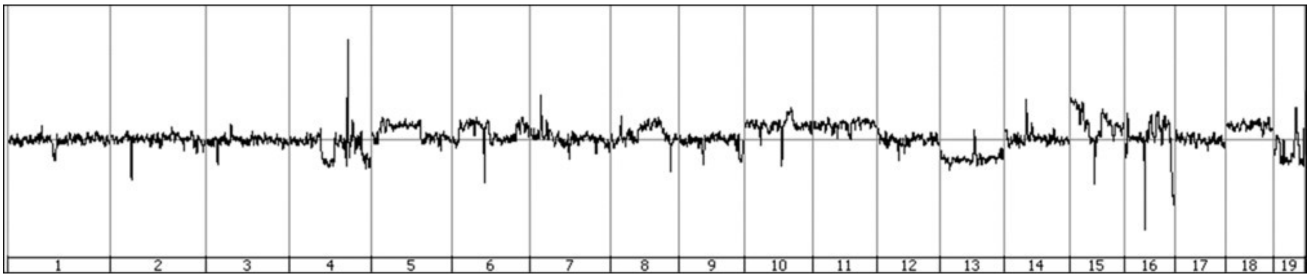

Figure S1 GL261 CTRL cell line genome view

The order of chromosomes is from left to right. Alterations in gain (plotted upwards) and loss (plotted downwards) compared to the reference genome (midline) are visible. Note the copy number oscillation of chromosome 4 and the copy number in gain for chromosomes 5, 6, 8, 15, 16, and 18. Data obtained by array CGH were analyzed by Agilent Genomic Workbench v7.0 software.

Figure S2

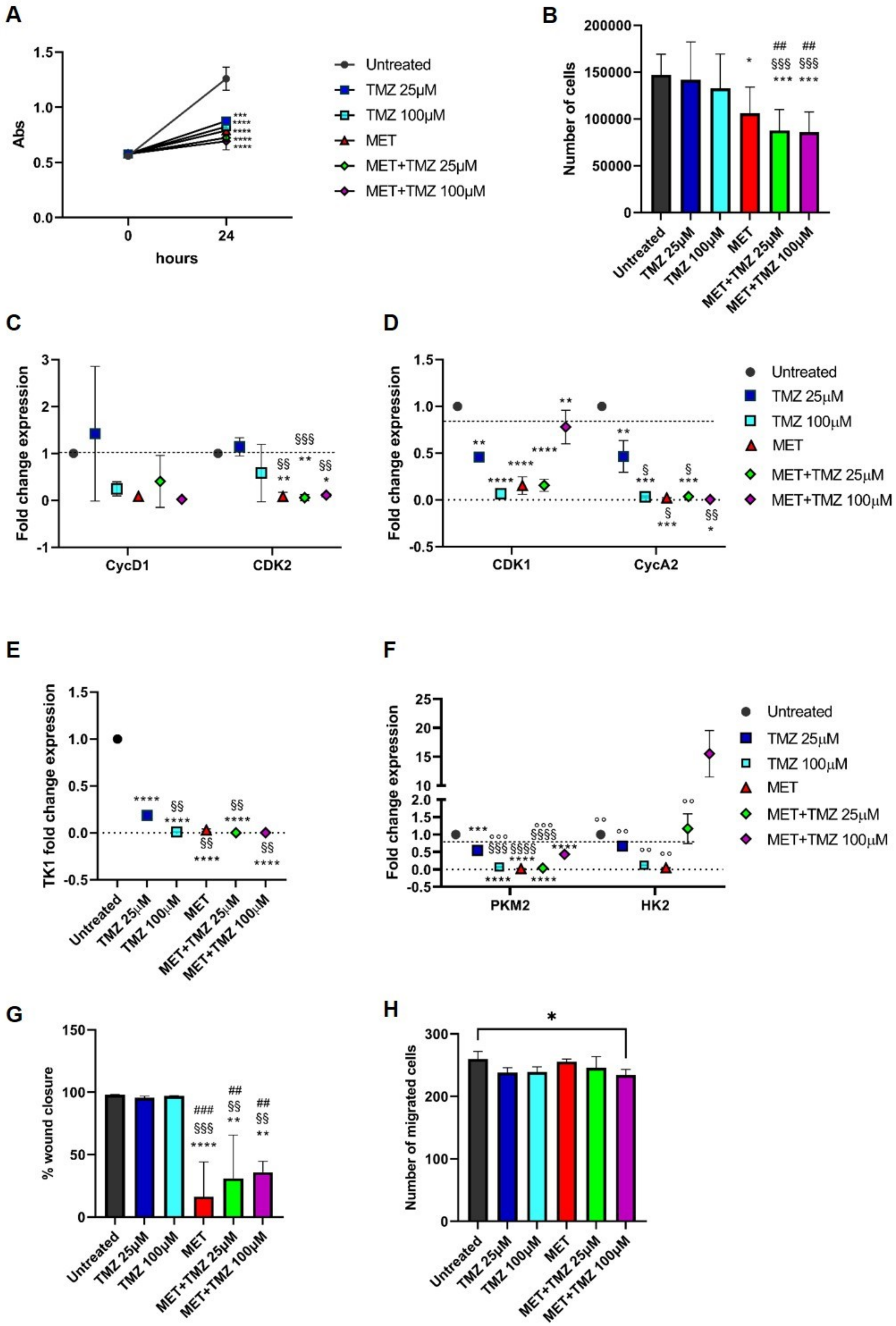
A
Abs
hours
Untreated
TMZ 25µM
TMZ 100µM
MET
MET+TMZ 25µM
MET+TMZ 100µM
B
Number of cells
Untreated
TMZ 25µM
TMZ 100µM
MET
MET+TMZ 25µM
MET+TMZ 100µM
C
Fold change expression
CycD1
CDK2
D
Fold change expression
CDK1
CycA2
Untreated
TMZ 25µM
TMZ 100µM
MET
MET+TMZ 25µM
MET+TMZ 100µM
E
TK1 fold change expression
Untreated
TMZ 25µM
TMZ 100µM
MET
MET+TMZ 25µM
MET+TMZ 100µM
F
Fold change expression
PKM2
HK2
Untreated
TMZ 25µM
TMZ 100µM
MET
MET+TMZ 25µM
MET+TMZ 100µM
G
% wound closure
Untreated
TMZ 25µM
TMZ 100µM
MET
MET+TMZ 25µM
MET+TMZ 100µM
H
Number of migrated cells
Untreated
TMZ 25µM
TMZ 100µM
MET
MET+TMZ 25µM
MET+TMZ 100µM

Figure S2 MET add on increased the effect of TMZ on GL261 cells proliferation blocking cells in G1/S phase and G2/M phase and reduced the ability of cell migration.

A Treatment toxicity was evaluated using MTT assay after 24 h of treatment. One-way ANOVA analysis followed by Bonferroni's multiple comparisons test was performed, ***p < 0.001, ****p < 0.0001 vs untreated cells. Data were expressed as mean values ± SD. **B** Cell viability was assessed by means of a Trypan blue exclusion test and expressed as the number of viable cells after 24 h of treatment. One-way ANOVA analysis followed by Bonferroni's multiple comparisons test was performed, *p<0.05, ***p<0.001 vs untreated cells; §§§p<0.001 vs TMZ 25 µM; $^{\#\#}$p<0.01 vs TMZ 100 µM. Data were expressed as mean values ± SD. Fold change expression ($2^{-\Delta\Delta CT}$ ± SD) revealed by RTqPCR of CycD1 and CDK2 (**C**), CDK1 and CycA2 (**D**), TK1 (**E**), and PKM2 and HK2 (**F**) genes treated for 24 h with TMZ 25 µM or TMZ 100 µM alone, MET 5 mM alone, MET 5 mM + TMZ 25 µM and MET 5 mM + TMZ 100 µM. Real-time PCR was performed in duplicate for each data point. One-way ANOVA analysis followed by Bonferroni's multiple comparisons test was performed, *p<0.05, **p<0.01, ***p<0.001, ****p<0.0001 vs untreated cells; §p<0.05, §§p<0.01, §§§p<0.001, §§§§p<0.0001 vs TMZ 25 µM; °°p<0.01,°°°p<0.001 vs MET 5 mM + TMZ 100 µM. **G** Graph represented the percentage of wound closure after 96 h of treatment. Data were expressed as mean values ± SD. One-way ANOVA analysis followed by Bonferroni's multiple comparisons test was performed, **p<0.01, ****p<0.0001 vs untreated cells; §§p<0.01, §§§p<0.001 vs TMZ 25 µM; $^{\#\#}$p<0.01, $^{\#\#\#}$p<0.01 vs TMZ 100 µM. **H** Graph of the number of migrated cells after 24 h of the treatment indicated Data were expressed as mean values ± SD. One-way ANOVA analysis followed by Bonferroni's multiple comparisons test was performed, *p<0.05 vs untreated cells.

Figure S3

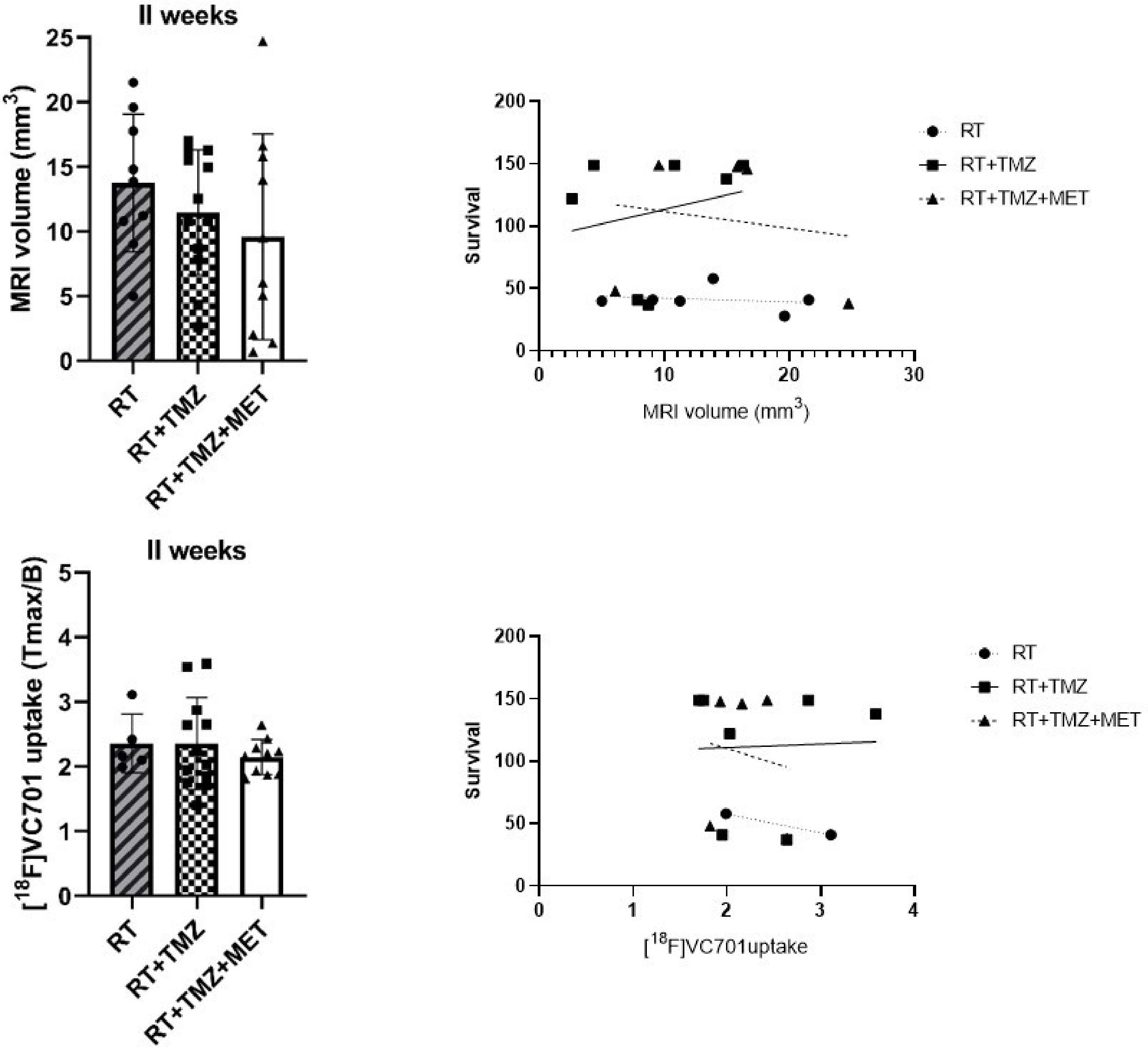

Figure S3 In vivo effect of radiotherapy treatment on MRI and $[^{18}F]$VC701.

At 2 weeks there wasn't any significant difference in tumor volume measured at MRI among treatment groups. Also we didn't observe any difference of $[^{18}F]$VC701 uptake. Each symbol represents one animal, bars and error bars indicate group mean±sd. Correlation curves indicated that nor $[^{18}F]$VC701 PET or MRI volume measured at 2 weeks, correlated with overall survival for both RT plus TMZ (Pearson r=-0,986, R^2=0,972, p<0,0001) or RT plus TMZ and MET (Pearson r=-0,903, R^2=0,816, p=0,0356).

Figure S4

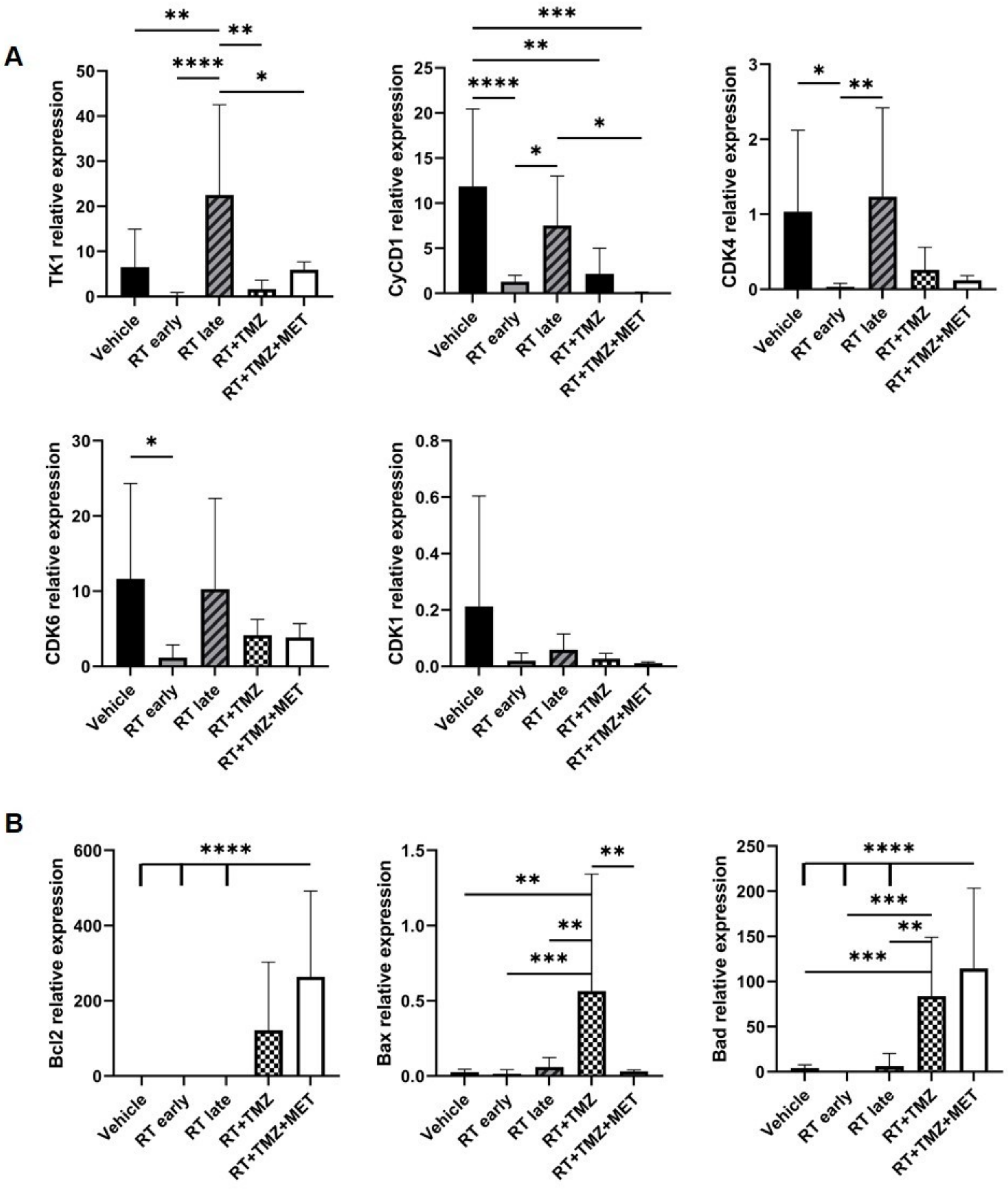

Figure S4 Ex-vivo effect of radiotherapy and drugs on cell cycle and apoptosis.

A Relative expression of RNA transcripts of TK1, CyCD1, CDK4, CDK6, CDK1 in GL261 tumor sample obtained from mice treated with vehicle (n = 4), RT early (n = 5), RT late (n = 4), RT+TMZ

(n = 3) and RT+TMZ+MET (n = 3). **B** Relative expression of RNA transcripts of Bcl, Bax and Bad in GL261 tumor sample obtained from mice treated with vehicle (n = 4), RT early (n = 5), RT late (n = 4), RT+TMZ (n = 3) and RT+TMZ+MET (n = 3). Gene analysis was performed in triplicate for vehicle and RT conditions and duplicate for RT + drugs. All replicates are used for the analysis. RT early mice were sacrificed 5 days after the beginning of radiotherapy whereas all the other mice were sacrificed at 28 days (4 weeks) from the beginning of the treatment. TK1: thymidine kinase 1, CyCD1: cyclin D1, CDK4: cyclin dependent kinase 4, CDK6: cyclin dependent kinase 6, CDK1: cyclin dependent kinase 1, RT: radiotherapy, TMZ: temozolomide, MET: metformin. Data are represented mean ± SD. * $p < 0.05$, ** $p < 0.01$, *** $p < 0.001$, and **** $p < 0.0001$ by ordinary one-way ANOVA analysis followed by Tukey’s multiple comparison test.

Figure S5

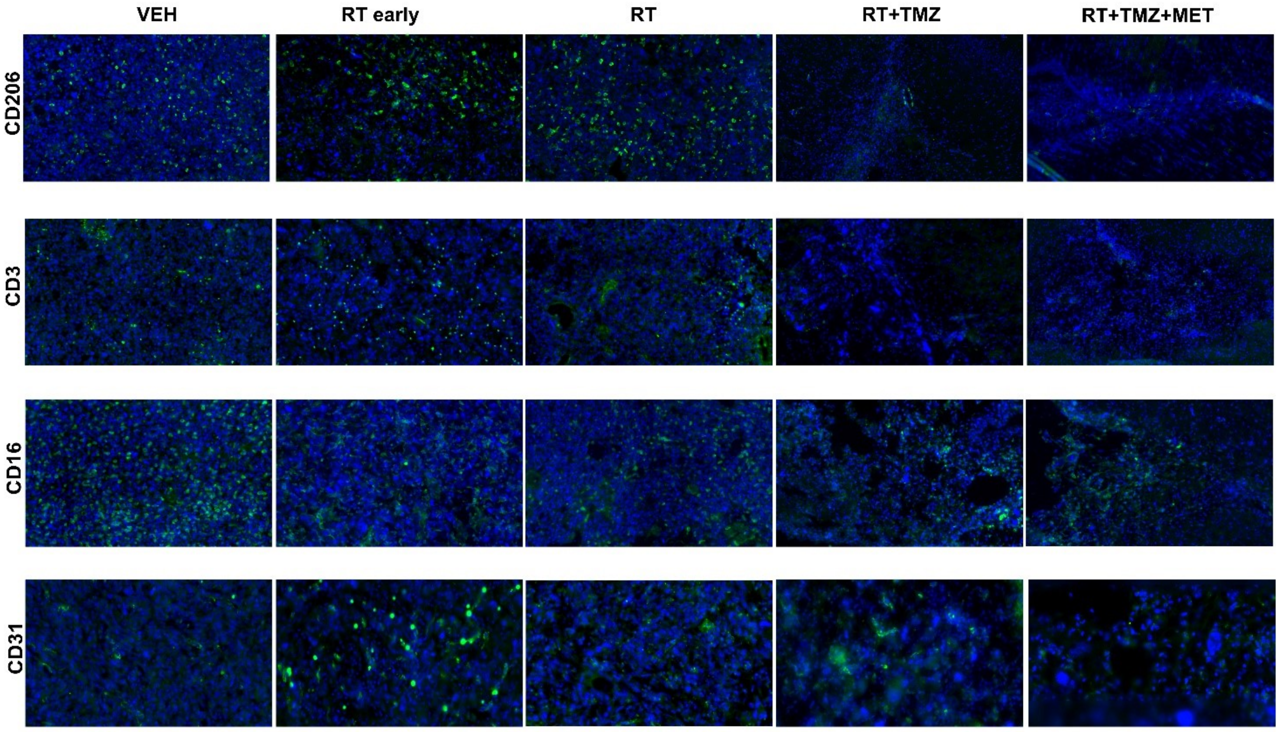

Figure S5 Post treatment expression of CD206, CD3, CD16, CD31

Immunofluorescence image of CD206, CD3, CD16, CD31 after treatment with vehicle, RT early, RT, RT+TMZ and RT+TMZ+MET.

Figure S6

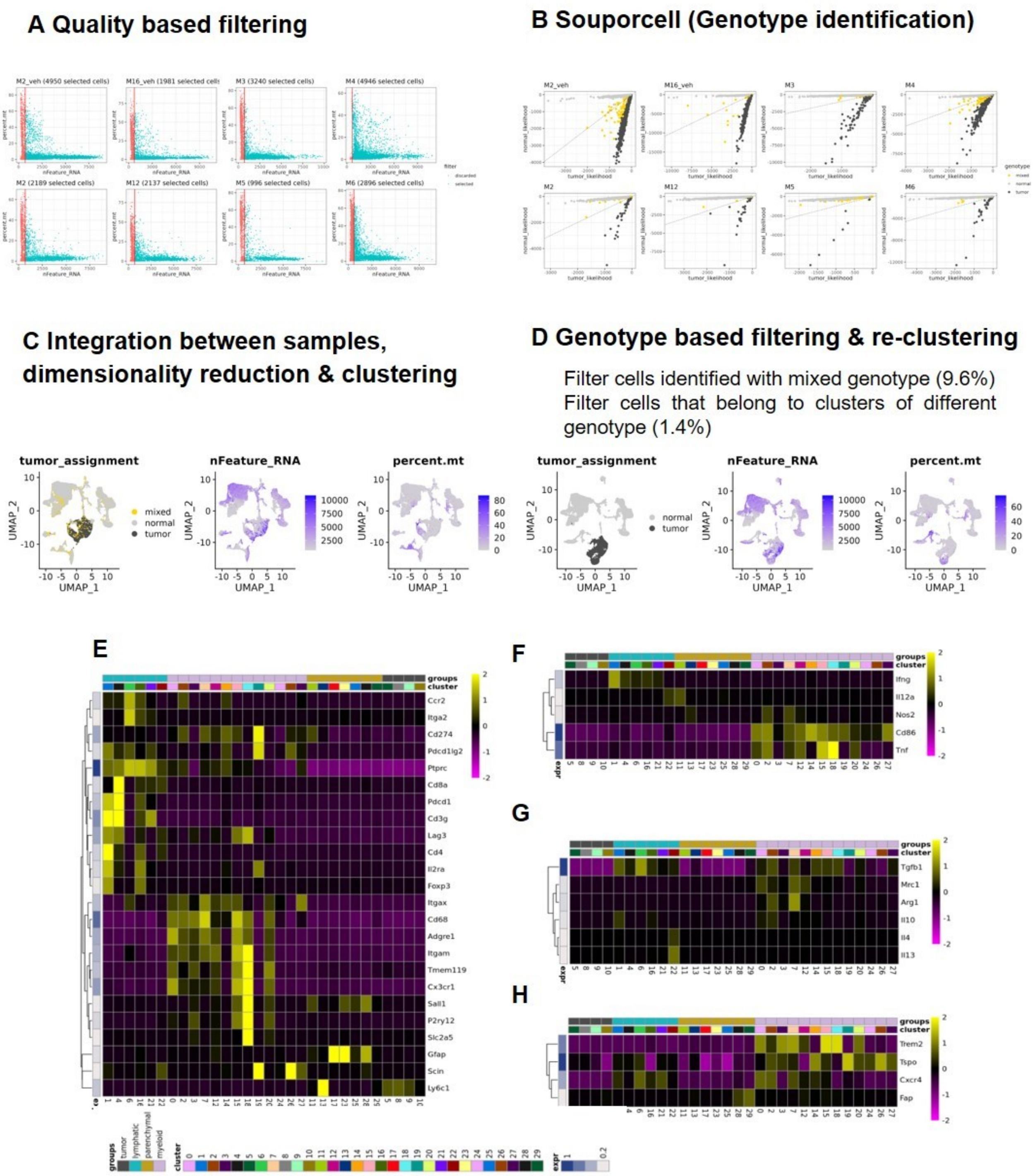

Figure S6 Schematic framework of data analysis and regions classification using a set of lineage associated transcript markers

A Cells with lower than 700 UMI counts were filtered out in each sample (26.7% of total cells). **B** Souporcell was employed to discern distinct genotypes within our dataset. Cells were segregated into

two genotypic categories within each sample, representing host mouse endogenous cells and orthotopic tumor cells. **C** Normalization, variable features identification and integration between samples was performed to produce a Uniform Manifold Approximation and Projection (UMAP) of the data. **D** Cells exhibiting mixed genotypes were removed to produce a final representation of the dataset. **E** Heatmap of specific transcript markers to perform a general classification; **F** Heatmap of transcript markers for M1- and M2-TAMs (**G**); **H** Heatmap of transcript markers of interest for tumor response to treatment.

122 **Figure S7**

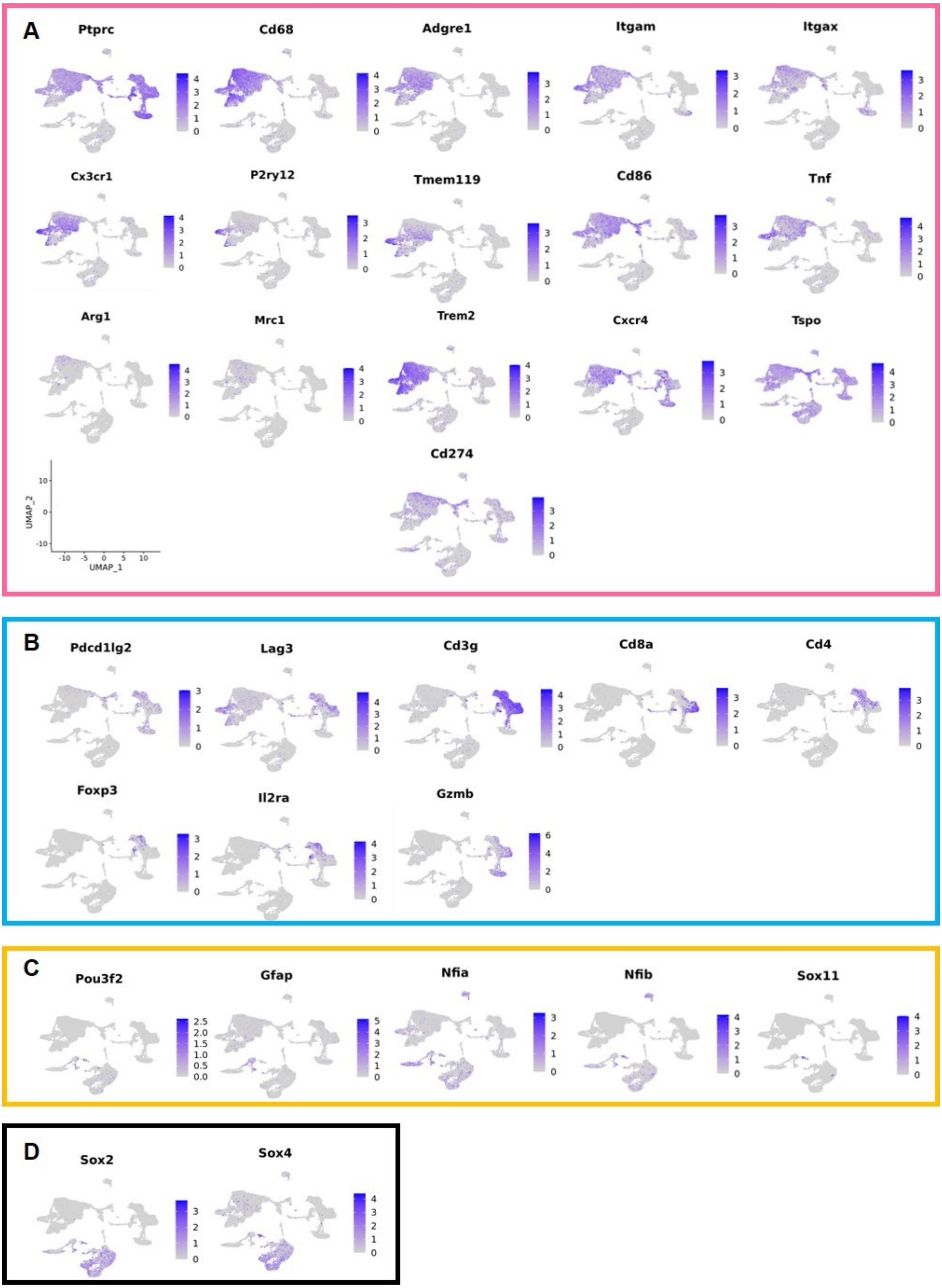

123

Figure S7 Distribution of gene expression level.

UMAP plots demonstrating the distribution of gene expression level for genes highly expressed by (**A**) myeloid population, (**B**) lymphoid population, (**C**) parenchymal population and (**D**) cancer stem cell population.

Figure S8

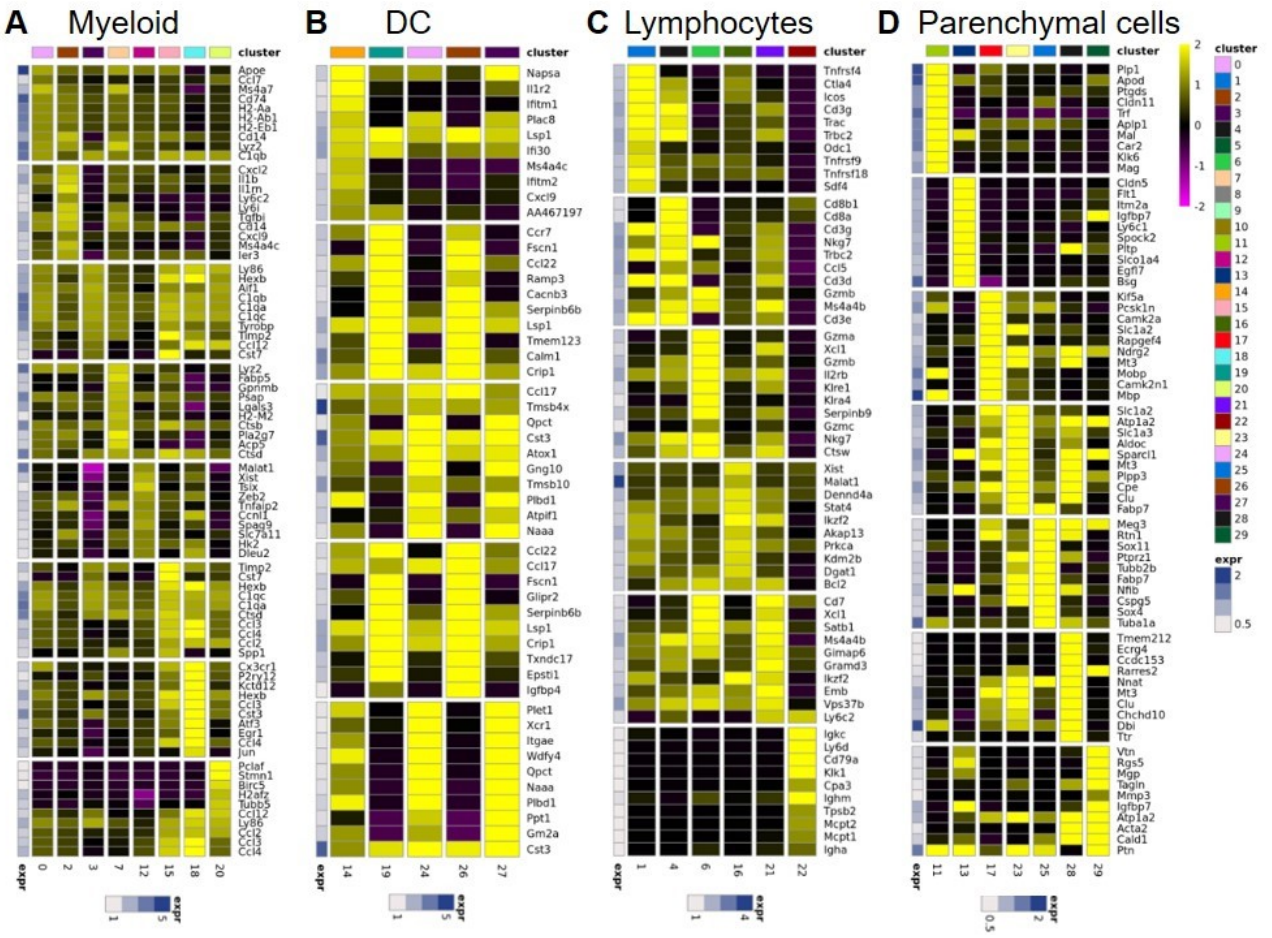

Figure S8 Non-tumor population cells are divided in different clusters. Heatmap of the first ten differentially expressed genes (DEGs) in (**A**) myeloid cells, (**B**) in dendritic cells, (**C**) in lymphocytes and in (**D**) parenchymal cells population.

Figure S9

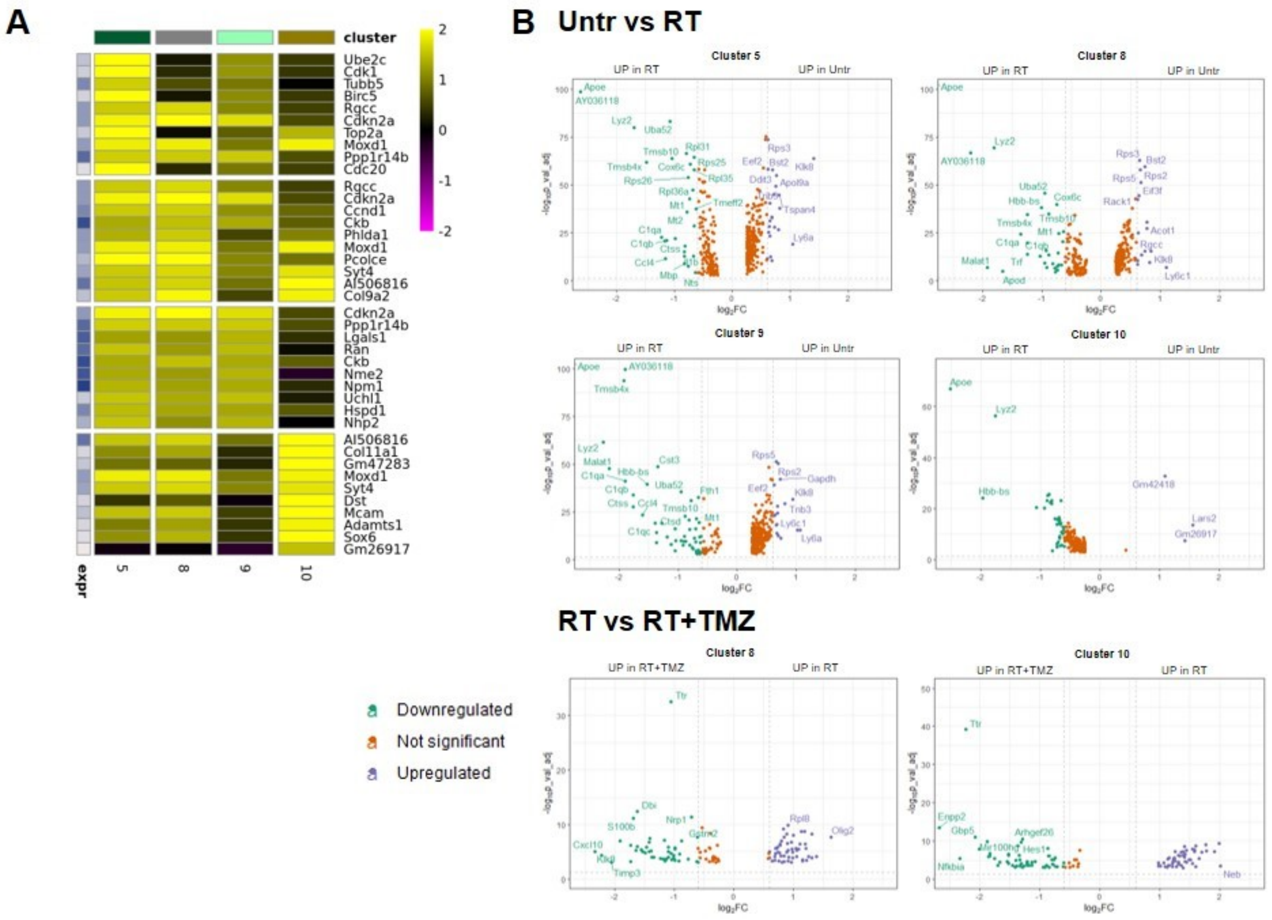

Figure S9 Tumor region included four clusters with heterogenous phenotype and different response to treatments. A Heatmap of the common differentially expressed genes in the four tumor subclusters: CL5, CL8, CL9 and CL10. **B** Volcano plots showing the results of DEG analysis between untreated versus RT and RT versus RT+TMZ treated tumors in the CL 5, 8, 9 and 10. Up-regulated genes of the first term of comparison are highlighted in blue and down-regulated genes of the first term of comparison are highlighted in green, and not significant genes are highlighted in orange ($Log_2FC > \pm0.3$; Adj $p<0.05$).

Figure S10

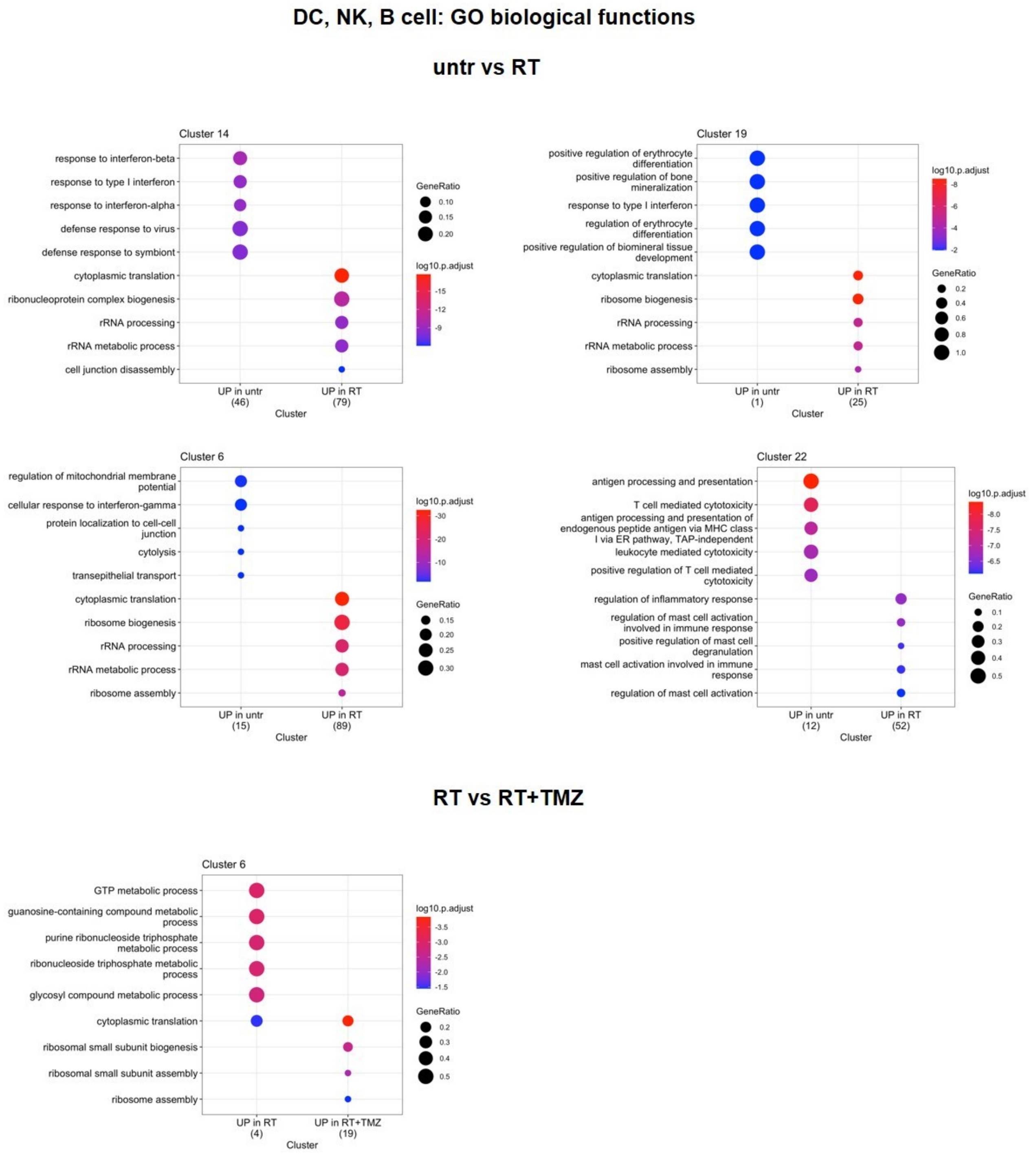

Figure S10 Modification in biological functions after therapy in T cells clusters

Top upregulated and downregulated biological functions in DC, NK, B cell clusters (14, 19, 6, 22) obtained by gene ontology (GO) analysis of significant DEGs previously identified among the groups: untreated versus RT alone and RT alone versus RT plus TMZ.

Supplementary table 1. Synteny between human Astrocytoma and Glioblastoma and copy number alteration at specific gene loci

Human Astrocytoma	synteny with mouse chromosomes	copy number alteration	comment
MYC 8q24.21	15qD1-qE1	this region is in gain (non mosaic)	gain of whole chr 15 (65% mosaic)
CCND2 (AMPLIFICATION) 12p13.32	6qF2-qG3	this region is in gain (60% mosaic)	no apmplification
CDK4 (AMPLIFICATION) 12q14.1	10qB5.3-qD3	this region is in gain (non mosaic)	no amplification
PDGFRA (AMPLIFICATION) 4q12	5qA3-qE3	this region is in gain (60% mosaic)	>70Mb
MET 7q31	6qA1-qB2.2	this region is in gain (60% mosaic)	>30 Mb
MYB 6q23.3	10qA1-qA4	this region is in gain (60% mosaic)	
**Human Glioblastoma**			
TERT	13qB3-qD2.3	this region is in loss (70% mosaic)	
EGFR [AMPLIFICATION] 7p11.2	chr.11	gain of whole chr 11 (78% mosaic)	no amplification
gain of whole chr 7	5qG3; 6qA1-qB2.2; 6qB2.2-qB3; 11	gain of regions syntenyc to human chr 7 (mosaic level 40-70%)	
PTEN 10q23.31	19qA-qC3	this region is in loss (60% mosaic)	
PDGFRA	5qA3-qE3	this region is in gain (60% mosaic)	>70Mb
MET	6qA1-qB2.2	this region is in gain (60% mosaic)	>30 Mb
FGFR3 4p16.3	5qA3-qE3	this region is in gain (60% mosaic)	>9,5 Mb
MDM2 12q15	10qB5.3-qD3	this region is in gain (60% mosaic)	
CDKN2A/CDKN2B	4qC4	this region is in gain (50% mosaic)	
BRAF	6qA1-qB2.2	this region is in gain (60% mosaic)	>30 Mb

Supplementary table 2: cell type and functional myeloid markers

	Peripheral myeloid			Microglia					Dendritic Cells			
	**0**	**2**	**7**	**3**	**12**	**15**	**18**	**20**	**14**	**19**	**24**	**27**
General												
Afi1 (IBA1)	++	+		+++		+	+	+++			+	+
Microglia homeostatic												
Hexb				+++	+	++++	++++	++				
Gpr34				+		+	++++	+				
Csf1r				+	++	++	++++	+				
Monocyte/MDSC												
Ly6c2		+++	+									
Ccr2		+							+			
M1												
Cd86	+	++	+		++	+	+		+	+		+
Tlr2	++	+++	+		++	++	+	+				
Tnf	+	++	+		++	+++		++				
M2 a,b,c,d												
Arg 1			++									
Mrc1 (CD206)	+		+									
Dab2	+	+	+		+	+		+				
APC												
H2-Eb1 (M2a,b)	+++	++	+	+	+				+++		++	+++
H2-Ab1 (M2a,b)	+++	+++	+	+	+				+++		++	
Cell cycle												
Mki67								++				
Pclaf								+++				
Stmn1								++++				
Invasion												
Ctsd	+		+++	+++	++	++++	++	+				
Cst7				+++		++++	+					
Spp1			+++	++		+++						

Timp2	+		+	+++		+++	++					
Angiogenesis												
Vegfa	++	++	++		++							
Cxcl2			++		++			+				
TAM												
Lyz2	+++	+++	+++<br>+		+							
Plac8		++++										
Trem2	+++		++	+++	+	+++	+++	+				
Tyrobp	+	+	+	+++		++	+	+				
C1qa	+++	+	++			+++<br>+	+++	++				
ApoE	++++	+	+++	+	++	++						
Fabp5			+++<br>+	++		++						
Gpnmb			+++<br>+			+						
Other markers												
Glul							++++					
Abca1					+++	+	+					
Malat1					++++							
Immunosuppression												
Cd274/PDL-1		+	+				+		+	+++		
Cd72	++	+										
Sirpa					++	++	++					
Lgal3			+++	++								
Lag3							++					

Manual annotation of cell type and phenotype markers of myeloid clusters. Significant differentially expressed transcripts (specific cluster versus all clusters included) indicated as: avg_log2FC <1 +; 1-1,5 ++; 1,6-2 +++; >2 ++++. General and microglial clusters already represented in Supplementary figure 7 and 8 are not included in the table. For Dendritic cells only functional markers are included

Table S3 Ligand – receptors markers

	Peripheral Mo			Microglia					Dendritic Cells					T Lymphocytes				Tumor			
Clusters	0	2	7	3	12	15	18	20	14	19	24	26	27	1	4	16	21	5	8	9	10
Cxcl2 (Cxcr2)																					
Cxcl9 (M/T)	+	+	+					+	++++												
Cxcl10 (M/T)		++				+		++	++									+	++		+
Cxcr3														+							
Cxcl13						+		++													
Cxcr5										+											
Cxcl16	++	++	++	+	+	+		+	++	+++		++	+								
Cxcr6														+++							
Ccl1														++++							
Ccr8														+							
Cxcl12 (++++ in C13)																					
Cxcr4	+	+	+																		
Ccl12	++	+		+++		++	++++	++++													
Ccl2	++	+		+	+	++++	++++	+++													
Ccr2		+							+					+		++					
Ccl3	++				++	++++	++++	+++													
Ccl4	++					++++	++++	+++													
Ccl5										++++		++++			++++		++				
Ccl7	++++					+	+	++													
Ccl8	++	+	++			++															
Ccr5	+		+		+		+														
Tgfb1		+					+		+												
Tgfbr1/2	+	+	+		+	+	+++								+						
Ilb1	++	++++	+			+															
Il1rn		++++	++		+	+															
Tnf	+	++	+		++	+++		++													
Tnfrsf			+	+		+	+							++++	+	+++	+				
Il6		+								+											
Il6ra	+	+			+		++														
Il10		+												+							
Il10ra/b	+	+	+		+	+	++														
Ifng														++++							
Ifngr T att						+	+		+	+				++	++	++	+++				
Mif				+														++	+	+++	
Cd74	+++	++	++	+	+	+			++		+		++								

Manual annotation of ligand – receptors transcripts from all clusters. Significant differentially expressed transcripts (specific cluster versus all clusters included) indicated as: avg_log2FC <1 +; 1-1,5 ++; 1,6-2 +++; >2 ++++. Receptors (in bold) are indicated after their ligands. Ilb is followed by its antagonist Il1rn.

Table S4 Cell type and phenotype T cell markers

**Clusters**	**1**	**4**	**16**	**21**
General				
Cd4	+++	+	++	
Cd8a		++++	+	+
Il2ra/Cd25	+++		+	
Rora			+++	
Tnfrsf4	++++		++	
Il7r		++	++	++
T activation				
CD27		++	++	++
Cd28	+++			
Icos	++++		+++	
Ifng	++++			
T suppression				
Ctla4	++++	++	+++	
Foxp3	+		+	
Hifa	+++			
Lag3	+++	++		
Cd279/Pdcd1	+++	++++	+	
Ikzf2 (Helios)	++++	+	++++	
Tox	++	+		

Manual annotation of cell type and phenotype markers of T cell clusters. Significant differentially expressed transcripts (specific cluster versus all clusters included) indicated as: avg_log2FC <1 +; 1-1,5 ++; 1,6-2 +++; >2 ++++.

Table S5 RT modulated transcripts

	Peripheral Mo			Microglia					Dendritic Cells		T Cell		Tumor			
**Clusters**	**0**	**2**	**7**	**3**	**12**	**15**	**18**	**20**	**14**	**19**	**1**	**4**	**5**	**8**	**9**	**10**
Ly6c2 (Monocytes)	----	---	----									+				
Isg15 (M1)	--	---	---	--		---			---	---			-		-	
Ms4a4c (M1)	--	---	---						---							
CD86 (M1)				++												
Cd74 (AP; Mif)		+						++				+			+	
H2-Ab1 (MHCII				++				++				+			+	
Cd81 (Invasion)		++		--		--		--								
Ctsd (Invasion)	++	+++	++						+++						++	
Cst7 (Invasion)				--				--								
Spp1 (Invasion)											+++					
Chil3 (TAM)		---	---													
C1qa (TAM)		+++									+	+	++	++	+++	+
Arg1 (TAM)	---	--														
Plac8 (TAM)	---	----	----						--							
Lyz2 (TAM)			+	++++	++++			++++			++	++	+++	+++	++++	+++
Tyrobp (TAM)														+	++	+
ApoE (TAM)	++	+++	++	+++		+++	++	+++	++++	++++	++++	++++	++++	++++	++++	++++
Trem2 (TAM)		-	++						+							
Gpnmb (TAM)	++		+			+++							-			
Cd72 (M. imm. Supr.)				--		----										
Lgals1 (M. imm. Supr.)								++								+
Lgal3 (M. imm. Supr.)			-					--								
Pdcd1 (T cell supr.)											+					
Ilb1 and Il1rn			--					++							++	
*Mif (**Cd74**)*						--										
*Ccl12 **(Ccr2)***								++								

*Ccl2 (**Ccr2**)*			--	--											+	
Ccr1	--	-														
*Cxcl2 (**Cxcr2**)*	--		---					+++					+	+	++	+
*Ccl4 (**Ccr5**)*													++	+	+++	+
*Ccl7 (**Ccr5**)*		+++														
*Ccl8 (**Ccr5**)*		+++	++			+++			+							
***Cxcr6** (Cxcl16)*											+	+				

Manual annotation of RT effects on function markers. Significant differentially expressed transcripts (RT compared with vehicle condition) indicated as: avg_log2FC <1 +; 1-1,5 ++; 1,6-2 +++; >2 ++++ for RT induced increase and <1 -; 1-1,5 --; 1,6-2 --; >2 ---- for RT induced decrement of transcript. Receptors are depicted in bold

Table S6 TMZ effects

	**Peripheral Mo.**			**Microglia**		**T cell**		**Tumor**		
Cluster	**0**	**2**	**7**	**3**	**12**	**1**	**4**	**5**	**8**	**10**
Cd74 (AP, Mif)			**+**						**++**	**++**
H2-Eb1 (AP)			**+**						**++**	**++**
Spp1 (Invasion)							**++**			
Arg (TAM)			**++**							
Malat1 (TMZ resistance)				**--**						**+++**
Cd274 (M. imm.supr.)	**+**	**+**	**+**							**++**
Pdcd1lg2 (M. imm.supr.)			**+**							
Lgals1 (M. imm.supr.)				**--**	**--**					**---**
Il18bp (M. imm.supr.)	**+**	**+**	**+**							
Cd4 (T cell)							**++**			
Cd8a (T cell)							**-**			
Pdcd1 (T cell supr)			**+**							
Tox (T cell supr)						**+**	**+**			
Cxcl10 (Chemotaxis)									**++++**	
Ccl8 (Chem. tum. Prog.)	**+**									
Ccl5 (Chemotaxis T cell)						**++**			**++**	
Ccr5 (Chemotxis T cell)							**+**			
Ube2c (Prolif.)				**--**						
Dbi (Lip. Met.)								**+++**	**+++**	
Syt4 (neur. Remod.)		**-**		**-**						
Ppp1r14b (imm. Infiltr.)	**-**			**--**	**--**	**-**			**--**	
Olig1 (neur. Marker.)									**--**	**---**
S100b (tum. Prog.)									**+++**	
Klk8 (tum. Prog.)									**++++**	

Manual annotation of TMZ add-on effect in comparison with RT alone on functional markers. Significant differentially expressed transcripts (TMZ+RT compared with RT condition) indicated as: avg_log2FC <1 +; 1-1,5 ++; 1,6-2 +++; >2 ++++ for TMZ induced increase and <1 -; 1-1,5 --; 1,6-2 --; >2 ---- for TMZ induced decrement of transcripts.

Table S7 Metformin effects

	Peripheral Mo			Microglia		T cell	
Cluster	**0**	**2**	**7**	**3**	**12**	**1**	**4**
Cd40 (AP)		-	-				
Cd74 (AP, Mif)	-	-	-				
H2-Eb1 (MHCII)	-	-	-				
Ctsb (Invasion)	-						
Cst7 (Invasion)	-		-				
Spp1 (Invasion)	+						
Lyz2 (TAM)	+						
Tgfbi (TAM)		-	-				
Tgm2 (Tcell migr)	-	-	-	+			
Gatm (tum. Prom.)	-	--	--				
Malat1 (TMZ resistance)					-		
Cd72 (Imm. Supr. M.)	-		-				
Lag3 (Imm. Supr. M.)			-				
Il18bp (Imm. Supr. M.)		-	-				
Cd4 (T cell)						-	----
Cd8a (T cell)							+
Lag3 (T cell supr.)						-	--
Ikzf2/Helios (T cell supr.)							-
Tox (T cell supr.)						-	-
Cxcr6 (Cxcr16) T cell. Supr.						-	-
Ccl6 (Ccr1) (Chemotaxis)			++				
Ccl3 (Ccr1) (Chemotaxis)							++
Ccl5 (Ccr5) (Chemotaxis)			--				
Ccl8 (Ccr5) Tum. Prog.	--	---	----	---			

Manual annotation of MET add-on effect in comparison with RT+TMZ alone on functional markers. Significant differentially expressed transcripts (MET+TMZ+RT compared with TMZ+ RT condition) indicated as: avg_log2FC <1 +; 1-1,5 ++; 1,6-2 +++; >2 ++++ for TMZ induced increase and <1 -; 1-1,5 --; 1,6-2 --; >2 ---- for MET induced decrement of transcripts.

Table S8 Sequences and primary antibodies used for the analysis

**Sequences**		
**Gene**	**Forward sequence (5’-3’)**	**Reverse sequence (5’-3’)**
14S	GGCAGACCGAGATGAATCCTCA	CAGGTCCAGGGGTCTTGGTCC
TK1	GGGGCAGATCCAGGTGATTC	GCATACTTGATCACCAGGCACTT
CycD1/CCND1	TGGATGCTGGAGGTCTGCGA	CGGCTCTTTTTCACGGGTCT
CDK2	CCTGGACACTGAGACTGAGG	GAATGACATCCAGCAGCTTGACAA
CycA2/CCNA2	ACTGCAAAGTTGAAAGTTTAGCAATGTT	GCCAGCTTTGTCCCGTGAC
CDK1	CACATGAGGTAGTAACACTCTGGTACAG	TGGTGCCTATACTCCAAATGTCAACTGG
PKM2	TACCATGCGGAGACCATCAA	AGCAACGGGCCGGTAGAG
HK2	CAAAGTGACAGTGGGTGTGG	GCCAGGTCCTTCACTGTCTC
**Antibodies**		
**Primary antibody**	**Host**	**Conditions**
IBA-1 (019-19741, Fujifilm Wako)	Rabbit	1:500; 4h RT
TMEM119 (ab209064, Abcam)	Rabbit	1:200; overnight at 4°C
GFAP (NB300-141, Novusbio)	Rabbit	1:100; overnight at 4°C
MMR/CD206 (AF2535, R&DSystems)	Goat	1:50; overnight at 4°C
PBR/TSPO (ab109497, Abcam)	Rabbit	1:250; 4h, RT
CD31/PECAM-1 (AB100-2284,Novusbio)	Rabbit	1:500; overnight at 4°C,
CD16/CD32 (#80366, Cell Signaling)	Rat	1:50; overnight at 4°C
CD3 (MAB4841-100, R&DSystems)	Rat	1:50; overnight at 4°C

Table S9 Number of animals included in the studies, grouped according to treatment conditions

**Survival**		
Conditions	***Number of animals***	
Vehicle	10	
MET	4	
TMZ	6	
TMZ plus MET	5	
RT	6	
RT plus TMZ	7	
RT plus TMZ with MET	5	
**Imaging studies**	**[^{18}F]FLT**	**[^{18}F]VC701**
Conditions	***Number of animals***	***Number of animals***
Vehicle	7	3
RT	9	5
RT plus TMZ	12	12
RT plus TMZ with MET	10	10

The number of animals employed in imaging investigations takes into account both radiotracers individually

Table S10 cell recovery after quality controls, processing and filtrations

A	cellranger filtered cells	nFeatures > 700	non mixed genotype	coherent clustering
untreated	9368	6931	6691	6652
RT	9510	8186	6763	6680
RT+TMZ	6361	4326	4137	4020
RT+TMZ+MET	6613	3892	3494	3448
Total	31852	23335	21085	20800

B	cellranger filtered cells	nFeatures > 700	non mixed genotype	coherent clustering
M2_veh	6230	4950	4766	4743
M16_veh	3138	1981	1925	1909
M3	4510	3240	3056	3024
M4	5000	4946	3707	3656
M2	3441	2189	2041	2000
M12	2920	2137	2096	2020
M5	1815	996	711	694
M6	4798	2896	2783	2754

Number of cells recovered and each passage for each condition. Part A) Cells deriving from Vehicle (n=2), RT (n=2), RT+TMZ (n=2) or RT+TMZ+MET (n=2) were pooled together. Part B) Cells deriving from each mouse: vehicle: M2, M16; RT: M3, M4; RT+TMZ: M2, M12; RT+TMZ+MET: M5, M6.
